# Deep-Sequencing Method for Quantifying Background Abundances of *Symbiodinium* Types: Exploring the Rare *Symbiodinium* Biosphere in Reef-Building Corals

**DOI:** 10.1371/journal.pone.0094297

**Published:** 2014-04-11

**Authors:** Kate M. Quigley, Sarah W. Davies, Carly D. Kenkel, Bette L. Willis, Mikhail V. Matz, Line K. Bay

**Affiliations:** 1 ARC Centre of Excellence for Coral Reef Studies, School of Marine and Tropical Biology, James Cook University, Townsville, Australia; 2 AIMS@JCU, Australian Institute of Marine Science and James Cook University, Townsville, Australia; 3 Department of Integrative Biology, The University of Texas at Austin, Austin, Texas, United States of America; 4 Australian Institute of Marine Science, PMB 3, Townsville, Queensland, Australia; Pennsylvania State University, United States of America

## Abstract

The capacity of reef-building corals to associate with environmentally-appropriate types of endosymbionts from the dinoflagellate genus *Symbiodinium* contributes significantly to their success at local scales. Additionally, some corals are able to acclimatize to environmental perturbations by shuffling the relative proportions of different *Symbiodinium* types hosted. Understanding the dynamics of these symbioses requires a sensitive and quantitative method of *Symbiodinium* genotyping. Electrophoresis methods, still widely utilized for this purpose, are predominantly qualitative and cannot guarantee detection of a background type below 10% of the total *Symbiodinium* population. Here, the relative abundances of four *Symbiodinium* types (A13, C1, C3, and D1) in mixed samples of known composition were quantified using deep sequencing of the internal transcribed spacer of the ribosomal RNA gene (ITS-2) by means of Next Generation Sequencing (NGS) using Roche 454. In samples dominated by each of the four *Symbiodinium* types tested, background levels of the other three types were detected when present at 5%, 1%, and 0.1% levels, and their relative abundances were quantified with high (A13, C1, D1) to variable (C3) accuracy. The potential of this deep sequencing method for resolving fine-scale genetic diversity within a symbiont type was further demonstrated in a natural symbiosis using ITS-1, and uncovered reef-specific differences in the composition of *Symbiodinium microadriaticum* in two species of acroporid corals (*Acropora digitifera* and *A. hyacinthus*) from Palau. The ability of deep sequencing of the ITS locus (1 and 2) to detect and quantify low-abundant *Symbiodinium* types, as well as finer-scale diversity below the type level, will enable more robust quantification of local genetic diversity in *Symbiodinium* populations. This method will help to elucidate the role that background types have in maximizing coral fitness across diverse environments and in response to environmental change.

## Introduction

Coral reefs are one of the most biodiverse ecosystems on earth [1], largely as a consequence of the symbiosis that exists between scleractinian corals and endosymbiotic dinoflagellates within the genus *Symbiodinium* [2]. The physiology and health of the coral host relies heavily on carbon translocation from these symbionts [3], [4], which enhances calcification of the coral host and leads to accretion of present day coral reefs [5]. The stability of this symbiosis is threatened by many factors, such as chronic and acute changes in CO_2_ [6], temperature [7], and irradiance [8]. These factors elicit stress responses from the coral holobiont (cnidarian host and associated dinoflagellate, bacterial and viral communities), causing the breakdown of symbiosis and loss of *Symbiodinium* from host tissues, a phenomenon known as bleaching [9]. Predictions of increased frequency and intensity of bleaching events represent a major threat to reef biodiversity and long-term viability of this important ecosystem [10], highlighting the need to fully understand the diversity and population dynamics of *Symbiodinium* types associated with corals.

Currently, nine genotypic clades are recognized within the genus *Symbiodinium* (A through I), with a range of types recognized within each clade (e.g. C1, C2, C3) [2], [11]. The relationship between sequence and physiological diversity of these dinoflagellates is still being investigated [12]. At least some coral species have been shown to harbor multiple *Symbiodinium* clades and types [13], [14] in abundances ranging from high (dominant) to low (background or rare) proportions of the endosymbiont community [15], [16], in associations that can vary in time and space. Uptake of novel types from the environment by adult corals (“symbiont switching” [2]) has received little experimental support (but see [17]), however, the relative abundances of pre-existing symbiont types can change substantially within the coral host as a result of environmental stressors (“symbiont shuffling”) [18], [19], [20]. These changes in *Symbiodinium* type complements can strongly influence holobiont fitness characteristics. For example, specific types within clade D (ITS-1 and 2) provide tolerance to higher temperatures [16], [18], [20], [21], C1 (ITS-1) can enhance coral growth rates [4], [22], type B2 (ITS-2) can enable recovery particularly rapidly after cold stress [23], and an unspecified type within clade A (ITS-2) confers high tolerance to elevated light [8]. Furthermore, holobiont physiological parameters can vary, not only with respect to the major clade of *Symbiodinium* hosted [21], [22], [24], [25], but also as a consequence of *Symbiodinium* type [26] and population within type [27]. The consequences of hosting functionally different clades, types and populations for coral physiology are increasingly appreciated [28], however, the full extent of this variation and its impacts on holobiont health and resilience remain open questions [29].

Standard techniques such as Single Strand Conformation Polymorphism (SSCP) and Denaturing Gradient Gel Electrophoresis (DGGE) are commonly used in *Symbiodinium* research to determine symbiont distributions or changes in dominant types after stress events [30], [31]. Briefly, SSCP and DGGE are gel-based electrophoresis techniques that allow for PCR products to be distinguished based on variation in nucleotide sequence [32], [33]. The dominant bands retrieved from DGGE accurately represent members of the *Symbiodinium* community in high abundance [34], and this coarse detection limit may make it immune to the accidental retrieval of non-symbiotic *Symbiodinium* found within the mucus or coral gastrovascular cavity. However, DGGE offers limited information about potentially crucial background *Symbiodinium* types due to poor detectability of low-abundant *Symbiodinium* types [16] and the generally qualitative rather than quantitative nature of this method. This has led to a major knowledge gap concerning the role of background types during ambient and stressful conditions [35], despite their potentially critical role in reef resilience during times of stress [36]. This is especially important as stressors such as increased temperature and CO_2_ may promote a change in the relative abundance of symbiont types through shuffling following bleaching [19]. Directional changes in relative symbiont abundances could change the holobiont's response to a bleaching stressor, representing relatively rapid acclimatization to increasingly stressful conditions. On longer time scales, symbiosis can also affect host speciation and niche diversification [37]. However, the extent to which *Symbiodinium* diversity and abundance influence a coral's capacity to adapt or acclimatize to warming and acidifying oceans is not yet fully understood and remains an area of active research [38].

Similar knowledge gaps concerning the role of low-abundance and/or uncultured marine microbial strains in other symbiotic organisms have been addressed using deep sequencing of DNA barcoded amplicons [39]. This research has highlighted the presence of core holobiont bacterial diversity and transient states associated with environmental stress [40], [41]. NGS in particular has uncovered vast assemblages of low-abundance bacteria, termed the rare bacterial biosphere, in the water column and associated with various marine invertebrate hosts and has elucidated the function of low-abundance bacteria within the holobiont, for example, in nitrogen cycling [42]. Although the detection of low-abundance microbes and *Symbiodinium* in corals has improved vastly with the much higher detection rate and quantitative accuracy offered by quantitative polymerase chain reaction (qPCR) relative to gel fingerprinting [15], [43], this method may still be biased by primer efficiencies and exclude symbiont diversity that is not recognized by specific priming sequences. While great promise is afforded by NGS methods, care must be taken to identify sequencing error and partition sequence variation within and among genomes (i.e., inter- and intra-genomic variants) to provide meaningful data on the distribution and abundance of *in hospite* and free-living *Symbiodinium*, especially those in low abundance. Cultured genetic stocks of *Symbiodinium* offer an opportunity to benchmark sequencing reads to cell numbers to reveal the presence of bias favoring dominant species [44] and its ability to distinguish real sequence variants from sequencing errors [45]. If quantitative and sensitive, NGS marker gene survey techniques [45] offer great potential for the detailed characterization of *Symbiodinium* diversity in time and space, including an examination of the rare *Symbiodinium* biosphere.

The utilization of the rDNA operon (which includes the ITS-1 and ITS-2 loci) has been pivotal to understanding *Symbiodinium* molecular diversity [46]. However, the high intra-genomic variation of this region and the tandem array of multiple ITS copies (multicopy) mean that assigning natural diversity is problematic [34], [47], [48]. For example, ITS-2 can be more variable, at times, between cells of the same *Symbiodinium* type than between types [48]. Furthermore, *Symbiodinium* types contain multiple and variable copy numbers of ITS loci, for example, clade D has an approximately 3-fold higher ITS-1 copy number than clade C (D: 3,181±69, [15]). Identifying real unique taxonomic units from variable intra-genomic copies exhibiting sequence variation remains a challenge in *Symbiodinium* research [34]. 454 NGS has been used to explore intra-genomic variation along the ITS-2 region for hundreds of plant species, and shown that only a few intra-genomic variants make up the vast majority (91%) of sequence reads retrieved per species [49]. NGS data may similarly allow for the inspection of intra-genomic variation within single types of *Symbiodinium* [50], however, further data are required before these analyses can be validated.

Here, we investigate the sensitivity and accuracy of marker gene survey techniques using the Roche 454 method to detect and quantify both dominant and background *Symbiodinium* types in mixed samples of known *Symbiodinium* type composition. This study also explores whether different symbiont clades and types are equally detectable using 454 NGS. We then demonstrate the potential of the new method for studies of natural coral populations by uncovering finer-scale, population-level differences in the genotypic composition of *Symbiodinium* within the same type hosted by corals from two reef sites in Palau.

## Materials and Methods

### *Symbiodinium* extractions

#### a. Algal cultures and cell counts for four Symbiodinium types

Four *Symbiodinium* sources (three cultured, one isolated from coral tissue) were used to assess the power of NGS to detect symbiont types and quantify their abundance. Three of these (ITS-2 types C1, D1 and A13) were cultured strains isolated from corals and grown in single-host inoculums in stock culture at the Australian Institute of Marine Science Algal Culturing Facility. They were genotyped using single-strand conformation polymorphism (SSCP) of ITS-1 with periodic genotyping using Sanger sequencing that confirmed their stability over time. Each culture was kept at 23°C and illuminated with 10,000 K PL-L 96 W fluorescent tubes (Catalina Compact), providing 60 µmol quanta m^−2^ s^−1^ photosynthetically active radiation (Li-Cor light sensor) for a 14∶10 (light∶dark) photoperiod. 30 ml of IMK media [51] containing *Symbiodinium* cells was transferred from bulk culture into new culturing flasks and kept for a period of 23 days, during which growth was monitored using a Multi-Well Spectrophotometer (Biotek) by measuring absorption at 700 nm. *Symbiodinium* viability was assessed using fluorescent microscopy (LEICA DM IRB). Cell density within each culture was quantified using a Neubauer Haemocytometer, with three replicates per symbiont type.

#### b. Cell counts for clade C3 and Symbiodinium extractions

*Symbiodinium* ITS-2 type C3 could not be maintained in culture and was therefore obtained from −80°C frozen coral samples previously genotyped using the electrophoresis-based method SSCP [52]. To extract symbiont C3 cells, coral tissue was separated from the skeleton using an air gun placed inside a plastic bag with 10 ml filtered sea water [53]. Tissue slurry was homogenized for 45 sec to disrupt muco-polysaccharides and filtered through a 20 mm mesh to remove coral skeleton. Samples were centrifuged at 3000 rpm for 2 min to pellet *Symbiodinium* cells. The cells were re-suspended in filtered sea water and pelleted three times and then re-suspended in 32 ml pure ethanol. Haemocytometer counts and dilutions for type C3 were prepared as described above for cultured material.

### Dilution series preparation and genomic DNA extraction

Cell densities were standardized to 1×10^6^ cells ml^−1^ for each of the four *Symbiodinium* types and fixed in ethanol. Samples of each *Symbiodinium* type were then thoroughly mixed and serially diluted, such that each of the four *Symbiodinium* types was present in samples at three densities, 0.1%, 1%, and 5%. Each of these densities was tested on the background of the three other types (Table 1). The combined cell density of all *Symbiodinium* types in each mixed sample was 1×10^6^ symbiont cells ml^−1^, with densities of each symbiont type varying according to Table 1.

**Table 1:** *Symbiodinium* cell dilution series showing the percentages of each type, ranging from 0.1% to 99.7% in each mixed sample and 100% in each pure type sample.^a^

	Known composition (%)								
Sample	A13	D1	C1	C3	PCR Cycles	Read #	Cleaned reads	Mapped reads	Mapping efficiency
1	99.7	0.1	0.1	0.1	23	1330	1330	1138	0.86
2	0.1	99.7	0.1	0.1	23	4215	4215	3663	0.87
3	0.1	0.1	99.7	0.1	23	15,019	1177	943	0.80
4	0.1	0.1	0.1	99.7	23	2335	2335	2151	0.92
5	97	1	1	1	23	12,591	12,591	10,762	0.86
6	1	97	1	1	22	5489	1943	1880	0.97
7	1	1	97	1	22	6105	9	7	0.78
8	1	1	1	97	23	1373	1373	1255	0.91
9	85	5	5	5	24	17,619	5574	3940	0.71
10	5	85	5	5	22	9868	9868	9264	0.94
11	5	5	85	5	23	11,266	11,266	9090	0.81
12	5	5	5	85	23	2410	2410	2186	0.91
13	100	0	0	0	24	11,911	11,911	8433	0.71
14	0	100	0	0	23	3760	3760	3599	0.96
15	0	0	100	0	24	10689	10,689	7048	0.66

a Combined *Symbiodinium* cell density for each sample was 1×10^6^ cells·ml^−1^.

Overall there were three replicate samples containing each clade at each background density (0.1%, 1%, 5%). Percentages correspond to the following volumes added: 100% = 1 ml, 99.7% = 997 µL, 97% = 970 µL, 85% = 850 µL, 1% = 10 µL, 0.1% = 1 µL. For example, in samples 2–4, *Symbiodinium* A13 comprised 0.1% of cells in each sample. PCR cycle number required to amplify each sample to roughly equivalent DNA concentration is given. The number of reads sequenced from the single 454 run (Read #), the number of cleaned reads after trimming and cleaning (Cleaned reads) and the final number of reads mapped for all clusters per sample (Mapped reads) are given.

Samples were prepared for genomic DNA extraction by centrifugation at 13,000 rpm for 5 min to remove any supernatant remaining from the dilution series preparation. DNA was extracted with a QIAGEN DNeasy Tissue Kit (QIAGEN) according to manufacturer's instructions and included an overnight Buffer ATL and proteinese K digestion at 55°C. Resulting DNA was quantified using a NanoDrop 1000 Spectophotometer (Thermo Scientific).

### Amplification of ITS-2 fragments and preparation for 454 sequencing

The approximately 350 bp ITS-2 region [32] was targeted and amplified in each sample to an equivalent concentration with 30 µL PCR reactions: 1× OptiBuffer (BIOLINE), 2 mM MgCl_2_ (BIOLINE), 2 mM dNTPs, 0.5 µM Forward (ITS2-F 5′-GTGAATTGCAGAACTCCGTG-3′) and Reverse (ITS2-R 5′-CCTCCGCTTACTTATATGCTT-3′) primers [54], 0.08 u·µL^−1^ BIO-X-ACT Taq (BIOLINE), and DNA template (30–50 ng). 13, 18, 23, 26, 29 PCR cycles with the following thermal profile were run for all samples: 3 min at 95°C, 30 sec at 95°C, 30 sec at 57°C, 30 sec at 72°C and 7 min at 72°C. The optimal number of PCR cycles was determined from 1% agarose gels as the first appearance of a faint band (22–24 cycles depending on the sample). The PCR products were cleaned using a PCR clean-up kit (QIAGEN), quantified using NanoDrop (Thermo Scientific) and diluted to 10 ng·µL^−1^. A second PCR was then performed to incorporate unique ITS-2 reverse barcodes and primers (Table S1 in File S2) for 454 sequencing with 15 samples (excluding sample 16, which was 100% C3) due to limitation in the number of barcoded primers. This second PCR contained ITS-2 primers at 0.5 µM (ITS2-F 5′-GTGAATTGCAGAACTCCGTG-3′) and ITS-2 Rapid barcoded Reverse primers (0.5 µM) with 1× OptiBuffer (BIOLINE), 2 mM MgCl_2_ (BIOLINE), 1 mM dNTPs, 0.08 u·µL^−1^ BIO-X-ACT Taq (BIOLINE) and DNA template (10 ng·µL^−1^). The ITS-2 454 PCR thermal profile for this second PCR followed: 4 cycles at 95°C for 5 min, 95°C for 30 sec, 59°C for 30 sec, 72°C for 1 min and 1 cycle at 72°C for 5 min. The resulting amplicons were visualized on an agarose gel to confirm equal amplification as described above. Differing volumes (13–20 µL) from visual comparisons of band brightness were used to pool uniquely barcoded samples. The pooled sample was precipitated using 0.1× (volume of the pooled sample) 3M Sodium Acetate, and 3× 100% EtOH then re-suspended in 25 µL nuclease-free water (note: the precipitation step is not necessary if proceeding directly to gel purification). The concentration of the pooled PCR product was measured using a NanoDrop photospectrometer (Thermo Scientific) and desiccated using a Savant DNA120 SpeedVac (Thermo Scientific) then shipped to the University of Texas at Austin (U.S.A). There, the pooled sample was run on an agarose gel in a single lane at 180 Volts for 20 min. The target 450 bp band was cut out and placed in 25 µL Milli-Q water overnight at 4°C, after which the eluate was sequenced at the Genome Sequencing and Analysis Facility at UT Austin.

### Quantifying *Symbiodinium* community composition with ITS-1 within field samples of corals

Tissue samples from *Acropora hyacinthus* and *A. digitifera* were collected from two sites in Palau (West Channel: N 07°31.557′ - E 134°29.428′ and Lighthouse: N 07°16.624′ E 134°27.619′) in 2009 under a Palau Marine Research Permit # PE-09-23. Three samples of each species were collected from each site (N = 12). Colonies sampled were >5 m apart to minimize sampling of clonal individuals. Tissue was stored in 96% ethanol and DNA was extracted following [55].

To further demonstrate the robustness of NGS sequencing across loci commonly used for symbiont identification, the ITS-1 locus, which is better characterized in Indo-Pacific *Symbiodinium* than ITS-2 [56], was used to determine symbiont diversity within *A. hyacinthus* and *A. digitifera*. The number of PCR cycles needed to amplify each sample followed the above protocol with the following changes: 0.075 U *ExTaq* Polymerase (Takara Biotechnology), 30 ng DNA template and 0.3 uM of forward (5′-CTCAGCTCTGGACGTTGYGTTGG-3′) and reverse (5′-GCTGCGTTCTTCATCGATGC-3′) primers of the approximately 330 bp ITS-1 region [33]. Cycle numbers ranged from 21–27 (Table 2). PCR products were purified using a PCR clean-up kit (Fermentas) and subsequent 454 barcoding followed the protocol described above for ITS-2 with the following changes: 0.025 U *ExTaq* Polymerase (Takara Biotechnology) and 0.3 uM unique 454 Forward ITS-1 Rapid barcoded primers (Table S2 in File S2). Sample preparation for sequencing was identical to the ITS-2 samples.

**Table 2:** Summary of twelve acroporid samples from Palau, their collection site, species identification, cycle number used to amplify fragment, total read number, number of mapped reads, mapping efficiency and their barcode for the 454 NGS.

Sample	Site	Species	Cycles	Read #	Mapped Reads	Mapping Efficiency
405	Lighthouse Reef	*A. hyacinthus*	Na	0	2543	na
406	Lighthouse Reef	*A. hyacinthus*	22	2634	2425	0.99
407	Lighthouse Reef	*A. hyacinthus*	25	2523	1037	0.99
499	West Channel	*A. hyacinthus*	25	1070	764	0.99
517	West Channel	*A. hyacinthus*	25	778	1262	0.99
518	West Channel	*A. hyacinthus*	25	1300	1287	100
2141	West Channel	*A. digitifera*	21	1331	1836	100
2183	West Channel	*A. digitifera*	23	1904	1697	0.99
2185	West Channel	*A. digitifera*	26	1761	1184	0.99
2211	Lighthouse Reef	*A. digitifera*	23	1221	1717	0.99
2214	Lighthouse Reef	*A. digitifera*	25	1773	1008	0.99
2215	Lighthouse Reef	*A. digitifera*	25	1052	2543	0.99

### Analysis of sequence data

The bioinformatics pipeline and analytical procedures presented here were custom written but follow current standards and approaches used in the field [45]. A workflow diagram, custom scripts and step-by-step data processing pipeline are described in full in File S1. The updated versions of the pipeline will be available at the Matz lab “Methods” web page, http://www.bio.utexas.edu/research/matz_lab/matzlab/Methods.html. The current version has been modified to accommodate sequence data from other NGS methods besides 454. All raw sequencing data has been deposited in the NCBI Sequence Read Archive under Accession number SRP038116.

#### a. Pre-processing

Raw .sff files from the sequencer were split by barcode, adaptor-trimmed including the removal of sequences corresponding to the PCR primers used. Nucleotide Quality Scores from the original .sff output file were filtered using the default Phred-equivalent score from the 454 Sequencing System Software Newbler program (v.2.6-05/2011, Roche/454). Sequences were also filtered for read length, and reads of less than 150 bp were discarded. Sample 2 of the dilution series (99.7% D1) did not meet quality control and was removed from the data set. Sample H405 from Palau failed to sequence and was not included in field sample analyses.

#### b. Clustering and selection of reference sequences

**Figure 1:**
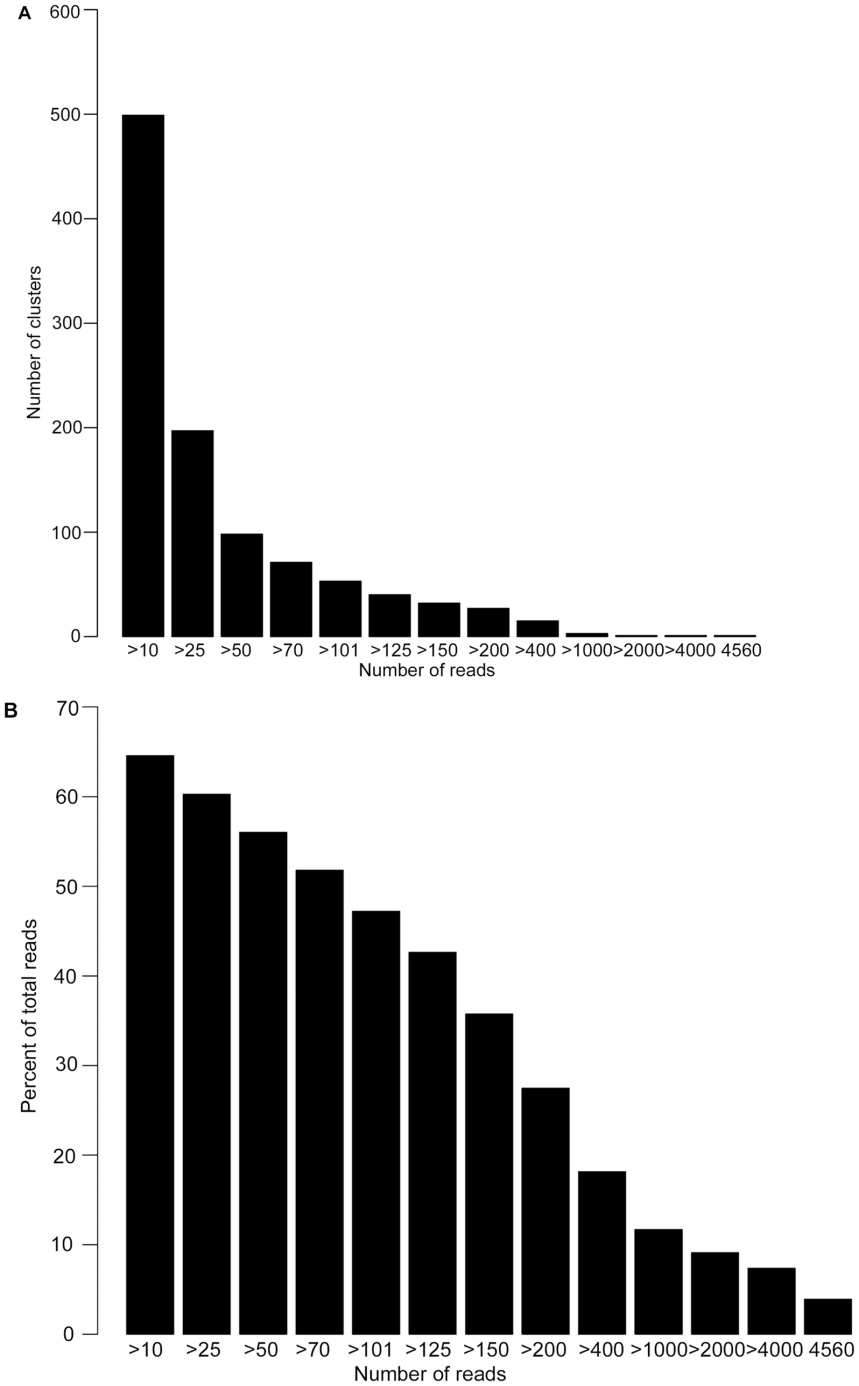
Histograms based on 100% identity clustering used to determine 454 NGS reference sequence cut-off. A. The number of identical reads assigned to each cluster. For example, there are approximately 500 clusters with at least 10 reads in each of those clusters. B. The number of reads clustered at each interval account for the relative proportion of reads across the total dataset. For example, clustering with at least 10 reads per cluster accounts for approximately 65% of the total reads in the dataset.

Reads that passed our quality filters were combined in a single FASTA file and clustered at 100% similar identity using Cd-Hit-454 (v.0.0.2, [57]). This method does not use prior information and clusters through repeated pairwise comparisons of reads among samples [45]. Clusters were grouped according to the number of reads per cluster (cluster size class), and patterns in the number of clusters per cluster size class interpreted from a histogram (Figure 1A). For each cluster size class, reference sequences were generated from Cd-Hit-454 by extracting marked sequences from the .clstr file. Some reference sequences were discarded to exclude unique read variants generated from PCR and 454 error. To identify such extraneous references, a second histogram (Figure 1B) comparing the percent of total reads retrieved per cluster size class was visually inspected. The optimal number of reference sequences to keep (the reference cut-off) was defined as the smallest number of references that accounted for the greatest percentage of total reads retrieved from the complete data set (i.e. an asymptote). No asymptotic relationship was found in our dilution data (Figure 1B), suggesting that all marked reference sequences retrieved from the .clstr file at each cluster size class should be used. Because of the known diversity in our data set, we were able to systematically explore this reference cut-off. Only by using all of the marked sequences (28) from the .clstr file as reference sequences (i.e., from those retrieved from >10 to 4560 reads per cluster) were we able to extract each of the four symbiont types added to our dilution series. Ultimately, we retrieved a total of 28 reference sequences.

As a second filter for 454 sequence artifacts, the reference sequences were screened for indels in homopolymer regions (but none were found), and overall pairwise similarity was computed using Geneious software (Biomatters Ltd., v.6.0). Quality- and length-filtered reads were then mapped against the reference sequences using the runMapping command of Newbler with the –rst (repeat score threshold) parameter set to 0 to achieve maximum sequence discrimination.

#### c. Annotation of reference sequences to clade and type level

The reference sequences were annotated to *Symbiodinium* clade level using blastn [58] matching against the NCBI “nr” database [59]. To develop criteria for discriminating between very similar ITS-2 sequences of C1 and C3 types [60], [61], we identified single-nucleotide polymorphisms (SNP) between NCBI sequences of C3 (HE579001.1) and C1 (JN558041.1 and HE578979.1). C212T was the only SNP discriminating *Symbiodinium* C1 and C3 database sequences (T in C3 and C in C1). All reference sequences identified by blastn as *Symbiodinium* C1 in our data had C at position 212, matching either C1 JN558040.1, JN558041.1 or EU106365.1 before editing, which is explained in more detail below (Table S3 in File S2). Remaining references, identified to clade C level by blastn (JN711498.1), shared a T at this position and were therefore annotated as *Symbiodinium* type C3. After annotation, the 28 reference sequences were categorized into: 17 A13 reference sequences [A13(c, i, j, k, l, m, n, o, p, q, s, t, u, v, w, zz, zzz)], four C1 references [C1(b, f, g, r)], three C3 references [C3(e, h, x)], and four D1 references [D1(a, d, y, z)]. The number of reads within a sample mapping to each reference sequence was extracted from the 454NewblerMetrics.txt.

#### d. Using the known dominant abundance samples to prune the reference library

The known abundance of *Symbiodinium* types in our samples offered a second check for estimating false positives in our reference library. Samples containing one *Symbiodinium* type in high (85–100%) abundance were expected to contain all reference sequences for that type, whereas samples containing *Symbiodinium* types in low abundance were expected to contain only a subset of all reference sequences per *Symbiodinium* type and no novel reference sequences. As predicted, all four D reference sequences were found in samples established as 97% D (Sample 6), 85% D (Sample 10) and 100% D (Sample 14), comprising 1836–8502 reads. To build the C3 library, all three reference sequences were kept from samples established as 99.7% C3 (Sample 4), 97% C3 (Sample 8), and 85% C3 (Sample 12), comprising 1182–2063 reads. Three of the four C1 references were kept from samples established as 99.7% C1 (Sample 3), 97% C1 (Sample 7), 85% C1 (Sample 11), and 100% C1 (Sample 15), comprising of 7–9090 reads. No reads from reference C1(g) were retrieved from the 97% C1 sample (Sample 7), therefore it was eliminated from the reference library. Samples established as 99.7% A13 (Sample 1), 97% A13 (Sample 5), 85% A13 (Sample 9) and 100% A13 (Sample 13) were used to build the A13 reference library, which was comprised of 1131–10,049 reads. Of the 17 A13 reference sequences, A13(i) was eliminated as no reads were retrieved from the 85% A13 sample (Sample 9).

#### e. Editing reference sequences and further annotation

After pruning, the resulting 26 reference sequences were aligned with Geneious software (Biomatters Ltd., v.6.0), and edited using BioEdit Sequence Alignment Editor (v.7.1.11). A type-specific consensus sequence was produced from the reference sequences, which was then edited as follows based on the consensus: 1) base pairs were changed to the consensus base pair when they differed at a given position, and 2) insertions or deletions were treated as single nucleotide polymorphisms [50], [61]. This approach did not materially change abundance estimates of type C1, C3 or A13, but collapsed the diversity of D into a single blast hit (D1) (Table S3 in File S2).

#### f. Assigning Symbiodinium type using blast

The top blast hit of all A13 reference sequences was to DQ174724.1 (90–91% maximum identity, Table S3 in File S2). The first ITS-2 blast hit was to type A13 (80–81% maximum identity). The four D reference sequences blasted with 97–98% maximum identity to JN558076.1 (D1). Types C3 and C1 reference sequences (3 each) blasted with 99% identity to JN711498.1 (clade C) and 98–99% to JN558041.1/JN558040.1 (C1) respectively. When maximum identity scores were equivalent between top blast hits, e-values were also used decide the reference sequence blast identity (Table S3 in File S2). Multiple reference sequences per type represented in the reference library may constitute: 1) intra-genomic variants, 2) rare, short-lived sequence variants 3) natural sequence variation at this locus within a single type that may or may not be temporally stable. The term haplotype was used to describe these sequence variants, which were treated as intra-genomic variants while acknowledging the possibilities described above. To explore intra-genomic variation and multicopy issues within the ITS-2 locus using 454 NGS, pairwise comparisons of haplotypes (i.e., within type reference sequence variants) were conducted using correlations among untransformed proportional data (percent) with the function scatterplotMatrix in the R package “car” [62].

#### g. Data quantification, normalization and statistical analysis

Haplotype networks were constructed in TCS (v.1.21) to visualize sequence variation amongst reference sequences that were annotated as the same *Symbiodinium* type [63]. The read counts per annotated reference sequence were extracted for each sample from 454NewblerMetrics.txt output files. To control for variable read depth among samples, the number of reads that were mapped to reference sequences were expressed as a percentage of the total number of mapped reads from that sample. Normalized counts for each symbiont type per sample did not conform with the assumptions of parametric tests, verified by using diagnostic plots in R (v.2.15.2, [64]). The mean observed value and non-parametric bootstrap confidence intervals (1000 bootstrap resamples) for each low abundance group (0–5%) were calculated using the R package Hmisc [65]. The non-parametric Gamma Rank Correlation Coefficient compared observed normalized reads to expected reads (based on cell mixing factor) for each symbiont type and its statistical significance based on 1,000 random iterations was calculated with the Rococo package in R [66], [67]. This test offers more robust significance testing than standard non-parametric correlation measures when data are variable [67]. To assess whether observed abundances were over- or underestimated at each of the expected abundance levels (0–5%) and across *Symbiodinium* types, residual plots were constructed in R from the linear regression of observed data.

For the field samples, linear mixed-effects models were run separately for each haplotype, with two fixed factors (species, site) and their interaction fitted to the arcsine square root-transformed, normalized reads of the dominant haplotypes. Assumptions of parametric pairwise testing of haplotype abundance were validated using diagnostic plots in R. Statistical significance of factors (Site, Species, Species x Site) was evaluated using likelihood ratio tests of nested models and if significant, a Tukey's test was used to evaluate pair-wise significance.

Haplotype networks were constructed to visualize sequence differences between the dominant haplotypes from the Palau field samples and their best blastn match using the Pegas package in R [68]. Insertions or deletions in reference sequences were treated as single nucleotide polymorphisms [50], [61]. To further evaluate how haplotype proportions vary between the two sites, proportions of the two dominant haplotypes were plotted against each other and site-specific patterns were visually assessed.

## Results

### Analysis of mixtures

Raw sequence output totaled 115,980 sequences (1330–17,619 sequences per sample), and 80,451 sequences remained after quality trimming (69.4%). Sequences grouped into 1038 clusters, which varied in size from clusters containing more than ten identical sequences (N = 499 clusters) to three clusters containing over 2000 identical sequences (Figure 1A). This latter pattern, of a few clusters containing a large number of reads, is consistent with expectations for dilution series samples that were dominated by one *Symbiodinium* type. A total of 65,359 reads unambiguously mapped across all clusters. Approximately 19% of quality-filtered reads (15,092) were discarded because of equally good mapping to more than one reference sequence (predominantly occurred between C3 and C1). A further 4,277 reads were discarded with the elimination of Sample 2 and haplotypes A13(i) and C1(g), leaving 61,082 sequences for further analysis. Of these 61,082 reads, 37.8% were mapped to 16 A13 reference sequences [A13(c, j, k, l, m, n, o, p, q, s, t, u, v, w, zz, zzz)], 20.5% to three C1 references [C1(b, f, r)], 14.7% to three C3 references [C3(e, h, x)], and 26.9% to four D1 references [D1(a, d, y, z)] across all samples.

#### 1. Detection limits for 454 NGS from mixtures

**Figure 2:**
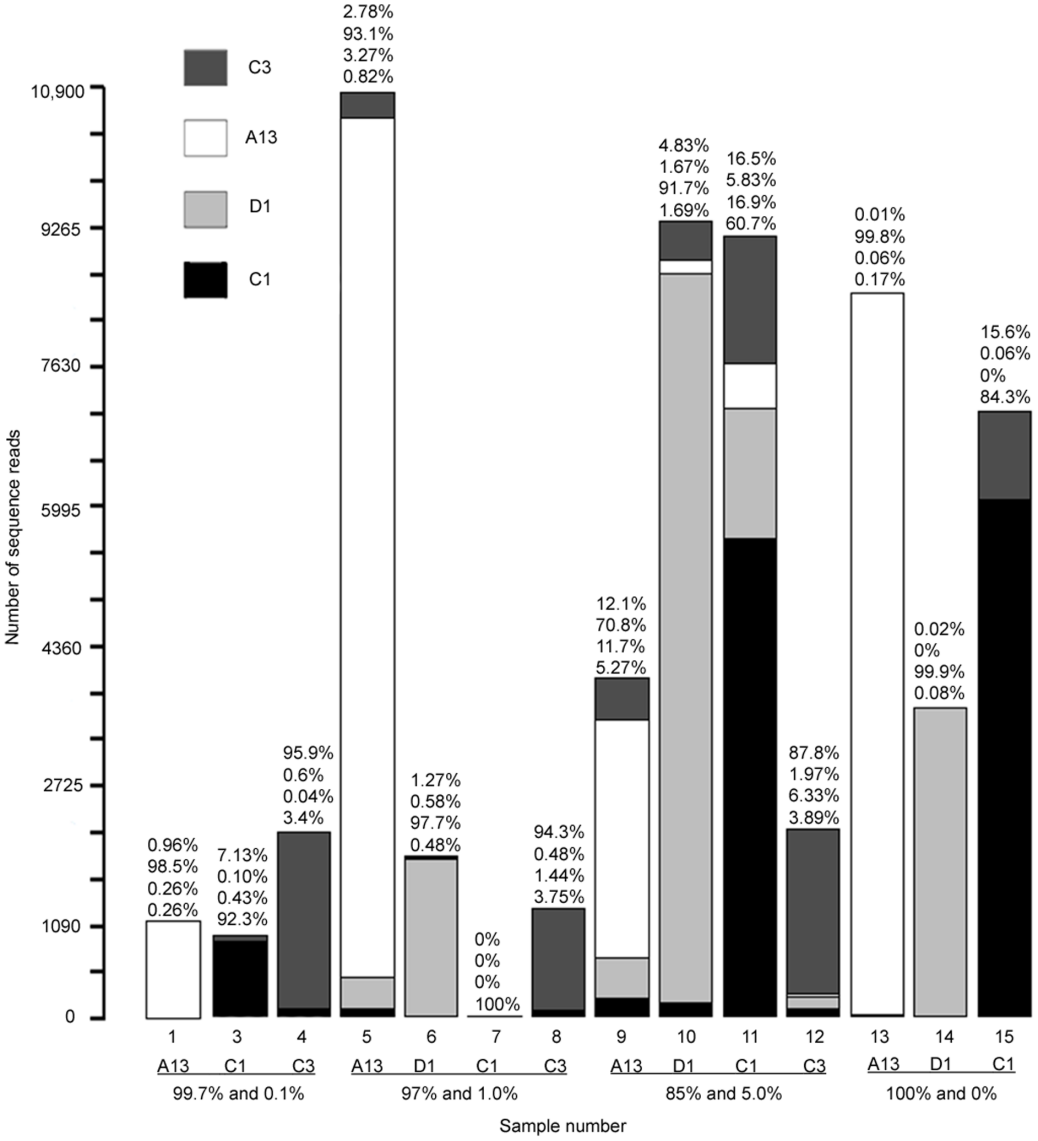
Number of reads mapped per *Symbiodinium* type in each sample. The percent abundance of each type quantified by ITS-2 454 NGS within the sample is listed above each stacked bar. Each percentage corresponds to the symbiont type listed in the figure legend in the top right hand corner, with the first percentage for each stacked bar being C3, followed by A13, D1 and C1. Sample numbers correspond to those found in Table 1.

454 NGS detected all symbiont types added to serial dilution mixtures, at densities ranging from 0.1% to 99.7% of mixtures, in each sample but one (i.e. Sample 7; Figure 2). In the pure 100% samples (13–15), NGS also identified sequences from the three other symbiont types at low (<0.05%±0.02% SE) background levels (range: 0.01–0.18% for C1, D1, A13), with the exception of C3 in sample 15. This outlier (sample 15) had 15.6% of all sequence reads mapping as C3 instead of the expected C1. In Sample 7, all sequences were identified as the dominant type C1 and none of the three background types added to mixtures at 1% were detected (Table 1; Figure 2), which most likely happened because this sample had the lowest number of reads after quality and length filtering (Table 1, Figure 2).

#### 2. Quantification of relative abundances of symbiont types in mixtures using 454 NGS

**Figure 3:**
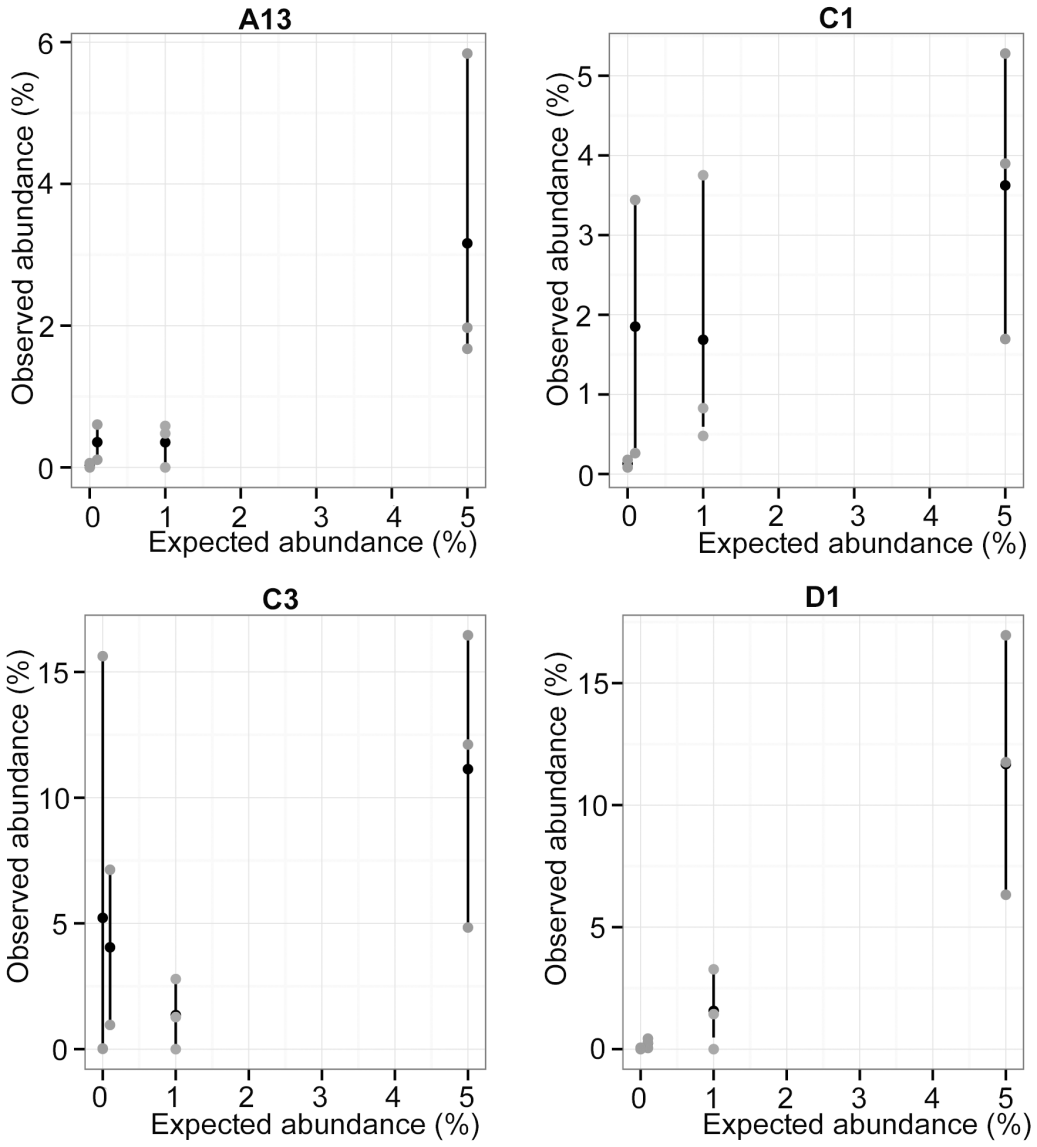
Observed versus expected background abundances (0.1–5%) for all symbiont types using ITS-2 454 NGS. Observed values were calculated by dividing the number of mapped reads retrieved per sample by the total number of reads retrieved for that symbiont type in that sample and multiplying by 100 (grey points). The mean value for each expected abundance and its 95% bootstrap confidence interval are shown in black. Background abundance estimates for symbiont types C1, A13, and D1 were strongly correlated to expected abundances (gamma = 0.76–0.90). However, the gamma correlation coefficient for C3 was 0.44, with a corresponding non-significant p-value.

Observed reads and expected cell proportions of *Symbiodinium* types were highly correlated when present in mixtures at background levels (0–5%) for symbiont types A13 (gamma = 0.78, *p*<0.016), C1 (gamma = 0.76, *p*<0.011), and D1 (gamma = 0.90, *p*<0.000). However, observed and expected proportions were not significantly correlated for low abundances of type C3 (gamma = 0.44; *p* = 0.16) (Figure 3). Furthermore, in addition to detecting the dominant symbiont type in pure samples (observed values: 84.3% C1, 99.9% D1, and 99.8% A13), C1 was also detected in two samples established without this type (at 0.08–0.2%; average 0.13%±0.04% SE), A13 was detected at 0.06% in one pure non-A13 sample, D1 was detected at 0.06% in one pure non-D1 sample, and C3 was detected in all pure non-C3 sample from 0.01–15.6% (average 5.22%±5.2% SE) (Figure 2). Residual plots showed no overall trend in either under- or overestimating abundances when types were present at lower abundance levels. For example, both 0.1% and 5% abundances showed equal division between over- and underestimated values. Overall, however, observed abundances were more frequently overestimated than underestimated. Comparing across types, *Symbiodinium* D1, C1 and A13 were all predominantly overestimated, while type C3 was underestimated when established at all abundances except at 1% (Figure S1).

#### 3. Haplotype diversity and intra-genomic variation

*Symbiodinium* type annotation, based on e-values and maximum identity scores from blastn, retrieved only one hit per reference sequence, but haplotype networks demonstrated finer scale variation between reference sequences (Figure S2). Reads were approximately evenly distributed across three D1 reference sequences (28–31.5%), while the fourth made up only 8.8% of the D1 reads. A majority (88.2%) of C1 reads were found in two reference sequences (b and f), with the remaining 11.8% of reads belonging to C1(r). Two C3 reference sequences collapsed into one haplotype using TCS algorithms, which encompassed over 96.8% of the reads retrieved for this type after mapping, and 3.14% of reads belonged to a second C3 haplotype. TCS also collapsed ten A13 reference sequences into one A13 basal haplotype making up 33.9% of all A13 reads retrieved, while six other haplotypes remained, one of which comprised 38.1% of all A13 reads.

**Figure 4:**
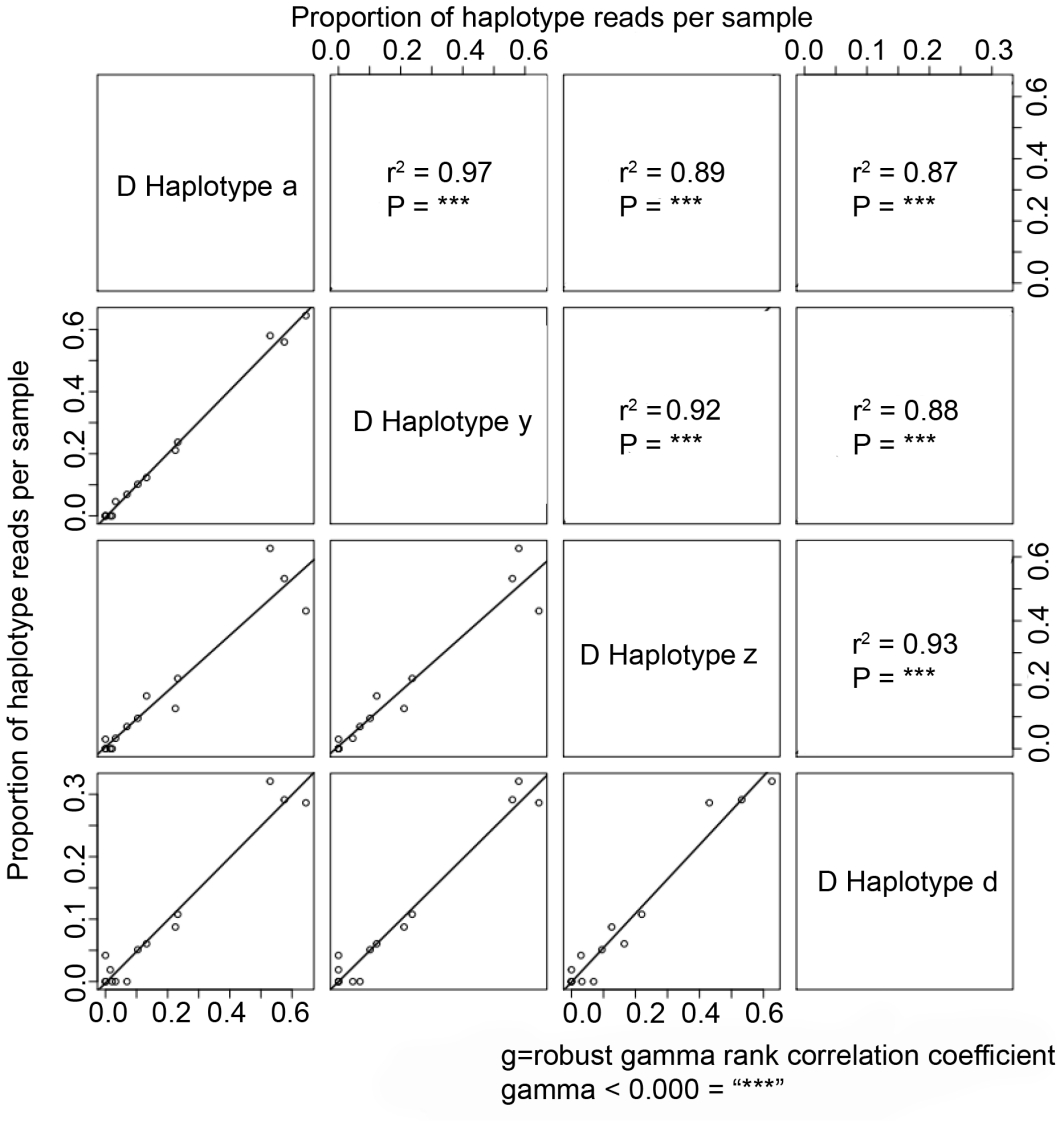
Multiple pairwise comparisons of the proportion of haplotype reads per sample for all four D1 haplotype reference sequences. Bold values in the upper diagonal represent Robust Rank Gamma Correlation Coefficients and their corresponding significance values.

To explore the assumption that all type-specific haplotypes represented intra-genomic variants resulting from the multicopy nature of the ITS operon [44], the proportion of reads per reference sequence type across all samples were pairwise compared with the expectation that intra-genomic variants should show positive correlation. All pairwise comparisons of the four D1 haplotypes were positively correlated (gamma>0.87, *p*<0.000, Figure 4), suggesting that the D1 nuclear genome may have four intra-genomic variants of the ITS-2 locus. Two of the three pairwise comparisons between C3 haplotypes were positively correlated (gamma>0.72, *p*<0.002) (Table S4 in File S2). This might suggest that this C3 type has two to three intra-genomic variants at this locus. Pairwise comparisons of the proportion of sequences retrieved of all three C1 haplotypes were significantly positively correlated (gamma = 0.77–0.84, *p*<0.003), again suggesting the number of intra-genomic variants to be at least three. Finally, scatterplots of the 16 A13 haplotypes showed strong positive correlations for all A13 haplotypes with the exception of 12 comparisons, of which only one was not significantly positive (gamma = 0.86, *p* = 0.06, Table S4 in File S2). To estimate the strength of A13 haplotype correlations, the proportion of sequences for the first reference (A13 haplotype c) were visualized and showed a positive correlation with all A13 haplotypes (gamma statistics >0.85, *p*<0.004, Table S4 in File S2). Although one set of haplotypes was not significantly positively correlated, it is unclear if this indicates distinct inter-genomic variants, given that the majority of haplotypes did show very strong positive correlations. Therefore, the majority of positive correlation values between A13 haplotypes suggest that this particular A13 type may have greater than 10 distinct intra-genomic variants. No negative correlations were found for any pairwise haplotype comparisons.

### 454 NGS as a method for detecting and quantifying *Symbiodinium* types within corals

#### 1. Output generated from 454 NGS for coral-associated populations of Symbiodinium

**Figure 5:**
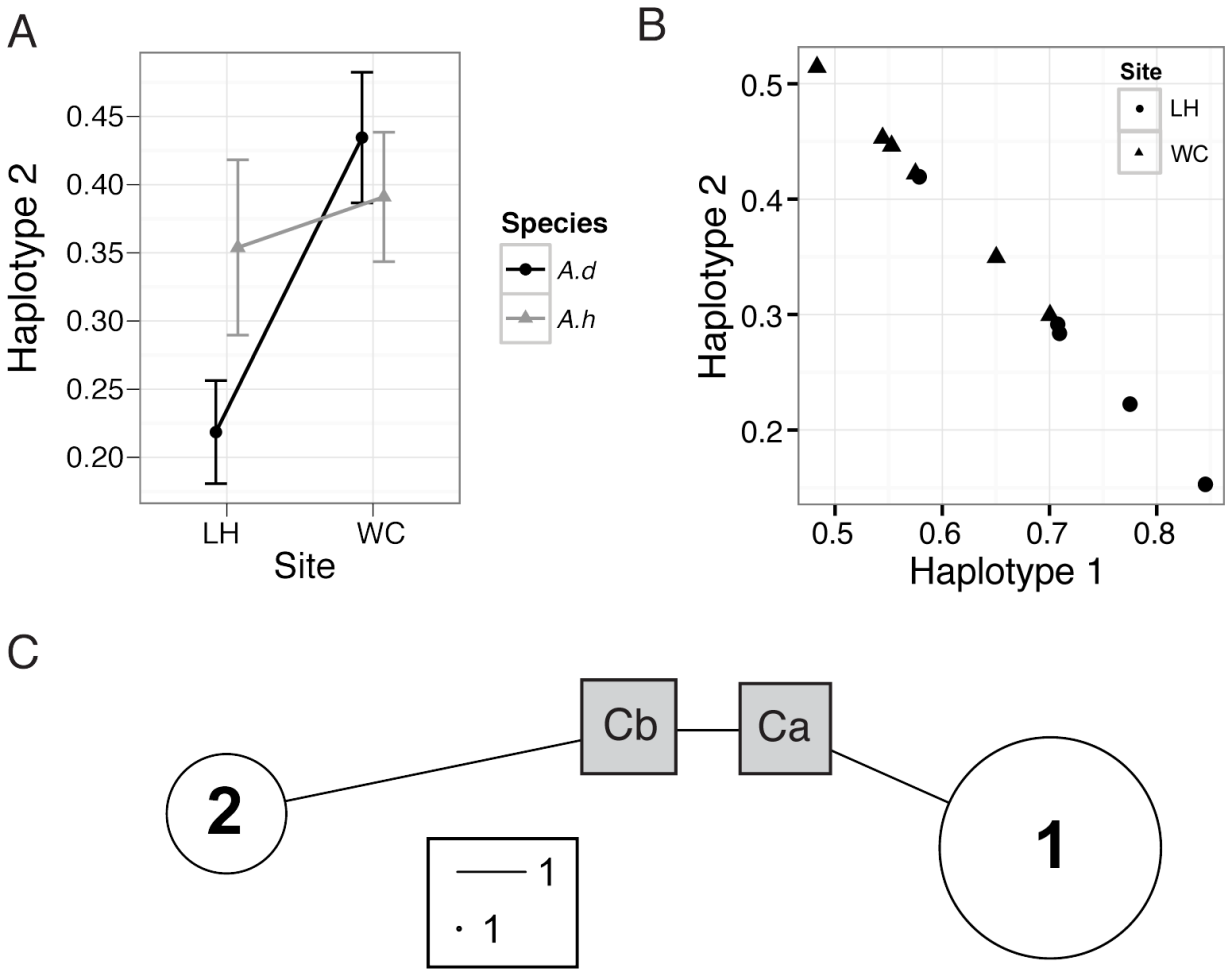
Results from ITS-1 454 NGS sequencing of *Symbiodinium* populations associated with *Acropora* colonies sampled from two sites in Palau. A. Variation in the proportion of haplotype 2 observed between two reef sites in Palau (Lighthouse reef (LH) and West Channel reef (WC)) for two species of corals, *Acropora digitifera (A.d)* and *A. hyacinthus (A.h)*. B. Regression showing how the relationship between the proportions of haplotypes 1 and 2 comprising *Symbiodinium* populations associated with *Acropora digitifera* and *A. hyacinthus* (n = 6 for *A.digitifera* and n = 5 for *A.hyacinthus*) varies among corals collected from two sites (WC and LH). C. Haplotype network for the two dominant haplotype clusters used as references visualized with two ITS-1 sequences from Genbank (Grey squares).

Raw sequence output generated from the 12 colonies of *Acropora hyacinthus* and *A. digitifera* from two field sites in Palau (three colonies per species from two sites) was 17,354, of which 16,773 remained after trimming, quality-filtering and mapping (Table 2). Two reference haplotypes were inferred (Figure 5C) and average mapping efficiency of samples was 99.8%. The number of mapped reads in each sample ranged from 764 to 2543 (Table 2).

#### 2. Symbiodinium diversity detected in acroporid corals from Palau

The two haplotypes mapped most strongly to clade C *Symbiodinium microadriaticum*, EU567166 (Ca, 1e-148) and EU567165 (Cb, 5e-179) (Figure 5C). Neither haplotype mapped perfectly to any references in Genbank but displayed very high sequence similarity. Haplotype 2 differed from Cb by only two SNPs and Haplotype 1 differed from Ca by two SNPs and a 3 bp deletion. A total of six mutations (SNPs and indels) distinguished the two haplotypes (Figure 5C).

A likelihood ratio test detected a significant species by site interaction (*p* = 0.045), with haplotype 2 comprising a lower proportion of the *Symbiodinium* complement in samples of *A. digitifera* than those of *A. hyacinthus* from Lighthouse reef (LH), but a similar proportion of the *Symbiodinium* complement in samples of both species from West Channel (WC) reef (Figure 5A). At both sites, there was a trend for haplotype 1 to be more abundant (LH: 0.72, WC: 0.58) than haplotype 2 (LH: 0.27, WC: 0.41), a trend that was statistically significant at Lighthouse Reef (Figure 5B).

## Discussion

The capacity to detect and quantify the abundance of *Symbiodinium* types associated with corals is essential for studies aimed at understanding holobiont physiology, susceptibility to stress and, ultimately, the resilience of corals to environmental change. Our results confirm that sequencing of the ITS-2 region using 454 NGS is able to detect the presence of co-occurring *Symbiodinium* types D1, C1, C3 and A13 at abundances as low as 0.1% of 1×10^6^ cells i.e., 1000 cells per sample. Amplicon sequencing of the ITS-1 region for *Symbiodinium* types associated with acroporid corals from Palau also demonstrated that this NGS approach can detect haplotype variants of *Symbiodinium microadriaticum* ITS-1 populations when *in hospite*, and distinguish differences in their frequencies among colonies and between sites that are less than 28 km apart. Our method is therefore well placed to detect and quantify rare, low-abundant haplotype variation within symbiont types that are likely under-represented by current methods of *Symbiodinium* detection (i.e DGGE and SSCP). We conclude that next generation sequencing will play an important role in providing a clearer understanding of microbial diversity and interactions between symbionts and marine metazoan hosts, including important groups like scleractinian corals.

454 NGS was able to quantify the abundances of types at low background levels (0–5%), whether they originated from cultured material or from freshly extracted DNA from frozen or ethanol-preserved tissue. This can be attributed to the 99.75% sequencing accuracy after clean-up and the high depth and coverage afforded by next generation sequencing [69]. For type C3, however, the degree of correlation between expected and observed background abundances was much weaker than for the three other symbiont types and not statistically significant. This may be due to high sequence similarity between C1 and C3, which led to more variable data for observed abundances (normalized read counts) compared to the other types. It is likely that small SNP PCR errors impact highly similar sequences differentiated by only one bp (as with C1 and C3 NCBI sequences), affecting clustering and/or mapping during bioinformatics processing and accounting for the detection of high numbers of C3 sequences in the pure 100% C1 sample. However, this method represents a significant improvement on those currently available for quantifying *de novo* background abundances of *Symbiodinium*.

Although no overall trend in inflation was found in residual plots from 0.1–5%, symbiont abundances were marginally inflated when they comprised 0.1% of cells in mixed *Symbiodinium* samples. Estimates for types C1 and C3 were 1.8–3.9% higher than expected, and those for D1 and A13 only 0.15–0.26% higher than expected. The largest deviations of observed from expected abundances occurred when C1 or C3 were in high abundance (for example, 60.7% C1 observed in a sample expected to be 85% C1). Such deviations were evident in most C1-dominated samples (3, 8, 11, 15), and especially in Sample 15, where 15.6% (1032 reads) of sequences were annotated as C3 in the 100% C1 sample. PCR or sequencing errors and uncertainty in clustering and mapping caused by high sequence similarity between C1 and C3 are likely to have contributed to these contradictory values, increasing the number of sequences identified as C1 at the expense of C3, as suggested by the C3 gamma correlation. Interestingly, our C3 population analyzed here may also include cells that contain sequences intermediary between those of other C3 and C1 populations, caused by the presence of both C3 and C1 sequences within the ITS-2 operon of this particular C3 population. Intermediary types, like C3h between C3 and C21, or type C3i between C3 and C1, have been documented and may have arisen through sexual recombination between the two types [70], [71], [72]. Hypotheses exploring the ecology and evolution of *Symbiodinium* can therefore be tested with NGS data.

### Detection of false-positives in pure samples

454 NGS detected low-abundant symbiont types in pure samples that were not expected in these single-type samples. Most pure samples returned 1–15 reads (4.83 reads ±2.1 SE), equivalent to 0.07%±0.02 SE, annotated as non-pure types. The exception was the C1 pure sample (Sample 15), which returned 1032 reads that matched C3 reference sequences C3 e (1.9%), h (81.8%), and x (16.3%). Unexpected reads may have occurred for a number of biological or technical reasons, including the presence of both C3 and C1 sequences within the same genome, contamination of other types or haplotypes that escaped SSCP detection during the genotyping of type C3 from frozen coral samples, clustering/mapping errors in bioinformatics processing or contamination at the cell culture or mixture stages. If we assume that the most likely explanation is that non-C3 reads found in pure samples signify contamination or PCR/454 error, the overabundance of C3 in Sample 15 (15.6%) becomes a biological outlier, which can be discounted. Accordingly, we propose a conservative detection limit cut-off at >0.11% ± two SEs (0.02).

### Read depth and coverage

The low number of sequence reads found for background types at expected abundances of 0.1% (1–74 sequences) raises questions about the number of sequences that are sufficient to confirm the identification of a symbiont type in a sample. Here, we use the depth of sequencing to discern the number of positive sequences required to parse out signal from noise and therefore set the detection limit of our assay. We set a 10,000 sequence minimum read number per sample for the mixed dilution samples, which would allow detection of minor types in 0.1% abundance with a coverage of 10. Nevertheless, some samples ended up with more or less reads representing minor types (1 to 74 reads). A single mapped read in a sample may be indicative of actual diversity; however, it is important to distinguish between single reads per sample and single reads across the whole data set. A single read in the data set with a unique identity (a unique haplotype or reference sequence) may equally represent a rare read variant (true diversity) or a PCR/sequencing error (false diversity). However, one read in a single sample may be more likely to represent true diversity if it is retrieved across multiple samples many times. Reads in our samples were only mapped to reference sequences if clusters had more than 10 reads in the combined dataset, thus eliminating singletons and many rare reads that had a high probability of being false positives. Robustness in detection may be increased by: 1) using biological replicates in the experimental design, 2) sampling greater than 1 million cells, i.e. more than one cm^2^ of coral tissue [73], or 3) sequencing at a higher coverage, albeit at an increased price per sample. These strategies will not only enable ecologically relevant distinctions in symbiont presence to be made, but will also increase detection of low abundance types.

### Quantifying sequence reads using NGS

A common issue in NGS marker gene surveys is how to relate read abundance with taxon abundance [44], [74]. The use of both multicopy and intragenomically variable loci for sequencing, in addition to biases associated with DNA extraction, PCR and 454 NGS, have led to a debate concerning whether read counts can be used for quantification purposes [44], [74], [75]. For example, NGS surveys of known dilution mixtures of fungal [44] and algal species [76] found order of magnitude differences in abundance estimates between species, and significant differences after filtering/clean-up steps in the number of reference sequences retrieved per species, however, intra-genomic variants or copy number were not accounted for in these studies. Alternatively, bacterial sequencing trials show 454 NGS to be both reproducible and quantitative [75], although some authors suggest that differences in read abundances between samples should only be compared within species [44]. It is likely that errors in quantifying type C3 in our study are related to copy number issues or sequence similarities with C1 at this locus. New computational methods employing locus copy numbers are now able to more accurately detect diversity and quantify species within environmental data sets [74].

### 454 NGS detects and quantifies fine-scale variation in *Symbiodinium* populations in hospite

Our results demonstrate the presence of variation in *Symbiodinium* diversity and population composition at much finer scales than previously detected. At the level of *Symbiodinium* type, symbiont diversity has been shown to vary with host species and biogeographic region, and in response to reef environment and depth [61], [70], [77], [78], [79], [80]. Detection of variation in *Symbiodinium* diversity between sites at the haplotype level, using a small sample size (N = 3 corals per site), highlights the levels of symbiont diversity the NGS approach is able to uncover. In addition, use of cloning in previous studies has restricted the number of sequences analyzed (e.g. [61]) compared to NGS. Differences in the proportion of symbiont haplotypes between the two reef sites, which are separated by ∼28 km, might reflect environmental specialization, perhaps to differences in wave exposure at the two sites (WC is more exposed than LH), indicative of local adaptation of the holobiont. Further research into variation in *Symbiodinium* population composition with environmental variation and manipulative experiments are needed to test this hypothesis.

### Detection and quantification thresholds for 454 NGS as compared to DGGE and qPCR

Two methods, DGGE and qPCR, are typically used to detect and quantify *Symbiodinium*; the former generally accepted as a non-quantitative technique [81], with detection thresholds of 10–30% of total symbiont abundance [30], [82], [83]. More recently, the high sensitivity of qPCR, which has 1000-fold greater detection ability than gel fingerprinting [15], [36], has made it a popular technique for detecting intra-clade types and for quantifying *Symbiodinium*. The detection limit for qPCR has been suggested to be roughly 7,000 cells per 1.5×10^6^ sample if using a single copy marker [36], equivalent to a 0.46% detection threshold. Despite this benchmark for detection, further experimental work is needed to determine the number of cells required for accurate and precise quantification of *Symbiodinium* abundance using qPCR because variability in amplification exists between clades D, A, B and C [36]. We did not detect amplification bias toward any clade or type with our NGS method, however, we did encounter bioinformatics challenges in separating types with high sequence similarity. With enhanced bioinformatics pipelines, sequencing using NGS has a greater capacity to detect and quantify *Symbiodinium* abundance when present at densities as low as ∼1,000 cells in 1×10^6^ (0.1%) than either qPCR or DGGE.

Finally, it is important to note that, unlike DGGE, 454 NGS does not appear to be a subjective technique. DGGE bands must be identified for each symbiont type and compared to other single bands or combinations of bands in adjacent lanes, introducing subjectivity in identifying their presence or absence. In comparison, the bioinformatic steps involved in NGS, which compare individual bases in each sequence using a standardized algorithm, remove such subjectivity. Furthermore, the sequencing data from NGS is able to differentiate intra-type variation (i.e. individual haplotypes) as well, as suggested by the numerous positively correlated haplotypes found for all symbiont types tested here. Thus far, only microsatellites have exhibited the ability to discern below the type level [84], however, specific microsatellites must be developed for most clades and types [52] and detection is limited to targeted loci, eliminating the possibility of finding novel diversity. The development of new loci for amplicon sequencing [85], possibly applied together with historically used markers such as ITS, will enable enhanced resolution to differentiate both clades and types. For example, the chloroplast DNA psbA^ncr^ locus is able to distinguish closely related types, but has limited resolution across clades [86]. The difficulty of differentiating between C3 and C1 in this study may therefore be ameliorated with the application of new and/or additional markers for NGS.

### *Symbiodinium* copy number and intra-genomic variation at the ITS-1 and ITS-2 loci

Many genes utilized for resolving *Symbiodinium* taxonomies are multicopy [16], possibly resulting from numerous complete and partial duplications of genes and genomes, as commonly seen in dinoflagellates [87], or through the integration of foreign DNA [88]. Attempts have been made to find single copy loci and determine copy number of known markers [85] and at least six other commonly used loci are multicopy: PsbA, Cp23S, 28S/5.8S, ITS-2, 18S [15], [89], [90], [91], [92]. For example, the actin region has seven copies in the clade C genomes tested and ∼1 copy in clade D genomes [16]. Pairwise correlations shown here suggest that the ITS-2 regions of types D1, C3 and C1 consist of multiple intra-genomic variants, results that may reflect the multicopy nature for the clades to which these types belong [16]. Therefore, finding a single-copy marker is essential to eliminate ambiguity stemming from *Symbiodinium* intra-genomic variability. This is particularly true in the context that ecologically relevant diversity likely exists on a continuum, from every read retrieved representing a unique haplotype (option 1), to the grouping of all read variants as intra-genomic variants of the same single symbiont type (option 2) [61]. The expected abundances presented here account for ITS-2 copy number by equating different reference sequences to represent intra-genomic variants, an approach that parallels option 1 of Stat and colleagues [61]. Further use of NGS data in conjunction with *Symbiodinium* genomic databases will play an important role in the identification and confirmation of intra-genomic variation across types.

As the mixture data presented here were of known diversity, read variants remaining after quality-control measures were assumed to be intra-genomic variants, and thus were pooled, enabling estimates of both diversity and abundances. However, the main challenges for applying this technique to natural samples with multicopy regions that exhibit intra-genomic variability will be: 1) distinguishing between variant sequences that represent intra- versus inter-species diversity; and 2) quantifying abundances. The use of NGS with a single-copy marker (and therefore no intra-genomic variation) that is able to detect equally well across *Symbiodinium* type diversity would clarify both detection and quantification problems. However, as ITS-2 is the predominant marker currently used to assign *Symbiodinium* diversity, intra-genomic variation in environmental data sets may be discerned using secondary structures and homology modeling [93], [94]. Indeed, the use of secondary structure analysis has been used previously in the construction of *Symbiodinium* and coral phylogenies [34], [95]. Developing more strategies in addition to pairwise comparisons [96] to account for *Symbiodinium* multicopy nature will improve both the precision and quantification of this method.

### Conclusions

In summary, this study is the first to evaluate the ability of NGS to quantitatively analyze samples with known densities of *Symbiodinium* types. We demonstrate here that NGS of *Symbiodinium* diversity is sensitive and quantitative, with a detection threshold at 0.11% of 1×10^6^ cells. Importantly, we also show that NGS is highly applicable for discerning haplotype-level diversity in natural coral populations. These results demonstrate that NGS has the potential to elucidate the diversity and abundances of background *Symbiodinium* types, either when endosymbiotic within coral hosts or possibly free-living in the environment. Further in-depth profiling of total *Symbiodinium* complements within host corals, now possible with this technique, will provide new insights into the relative abundance of *Symbiodinium*-type specialist and generalist corals [14], [97], and will enable the development of better models to predict host susceptibility to stress events. Our results demonstrate that this new methodology will significantly advance the evolutionary and ecological understanding of this important photosymbiont.

## Supporting Information

Figure S1 **Residual plot of standardized residuals calculated from the linear model of observed and expected abundances.**
(TIF)

Figure S2 **Haplotype networks for each** ***Symbiodinium*** **type constructed from edited reference sequences.** Gaps are treated as a fifth state in TCS.
(TIF)

Figure S3 **Workflow corresponding to single locus** ***Symbiodinium*** **454 Next Generation Sequencing bioinformatic pipeline.** *1) .sff to .fna, 2) map adaptors, trim and discard shorts, 3) convert back to .sff file incorporating this new information (trimmed .sff), 4) trimmed .sff to .fas, 5) Rename .fas to correspond to sample identities, 6) Group all renamed .fas files into one .fas file.
(TIF)

File S1 **Detailed pipeline for** ***Symbiodinium*** **454 Next Generation Sequencing data from a single locus (i.e. ITS-1 or ITS-2).**
(DOCX)

File S2 **Tables S1, S2, S3, and S4.**
(DOCX)
