# Supplementary figures and images for: Deep-Sequencing Method for Quantifying Background Abundances of Symbiodinium Types: Exploring the Rare Symbiodinium Biosphere in Reef-Building Corals
Source: PLoS One. 2014 Apr 11;9(4):e94297. doi: 10.1371/journal.pone.0094297 (PMC3984134; doi:10.1371/journal.pone.0094297)

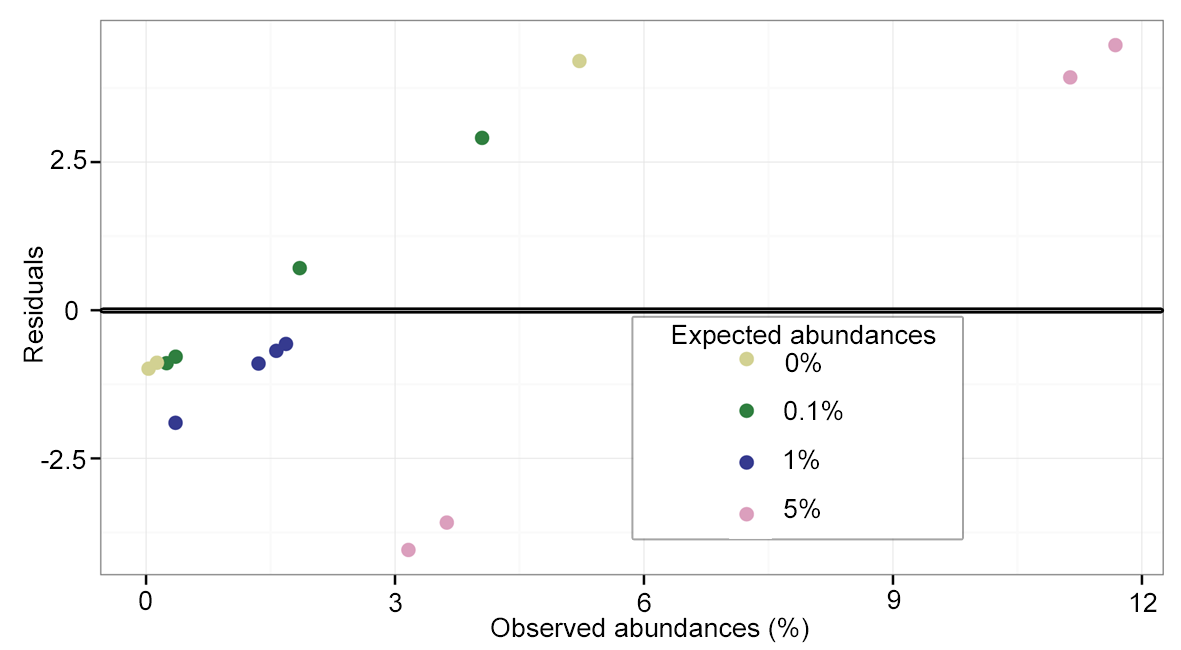

Supplement: Figure S1 — Residual plot of standardized residuals calculated from the linear model of observed and expected abundances. (TIF) [file pone.0094297.s001.tif]

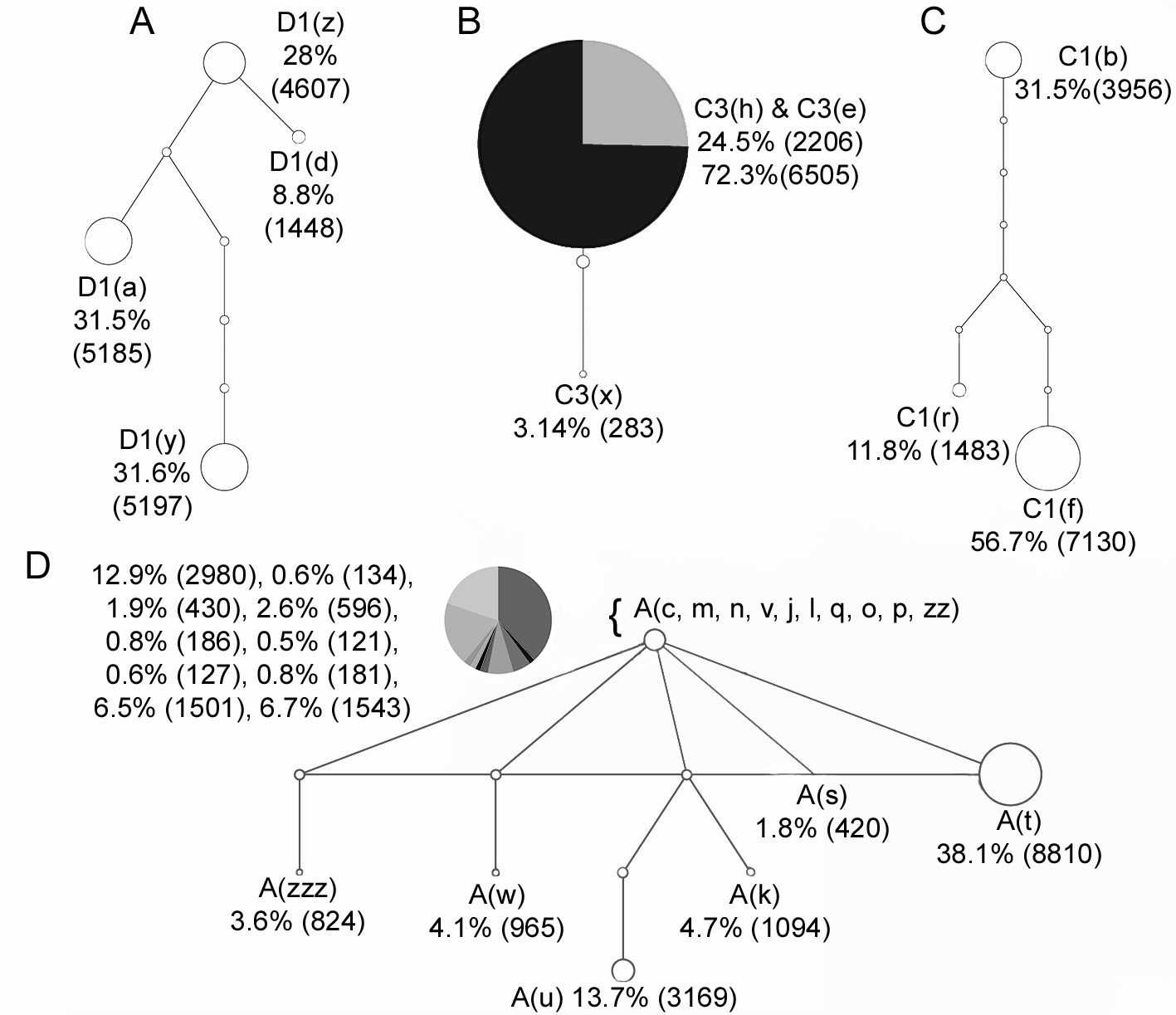

Supplement: Figure S2 — Haplotype networks for each Symbiodinium type constructed from edited reference sequences. Gaps are treated as a fifth state in TCS. (TIF) [file pone.0094297.s002.tif]

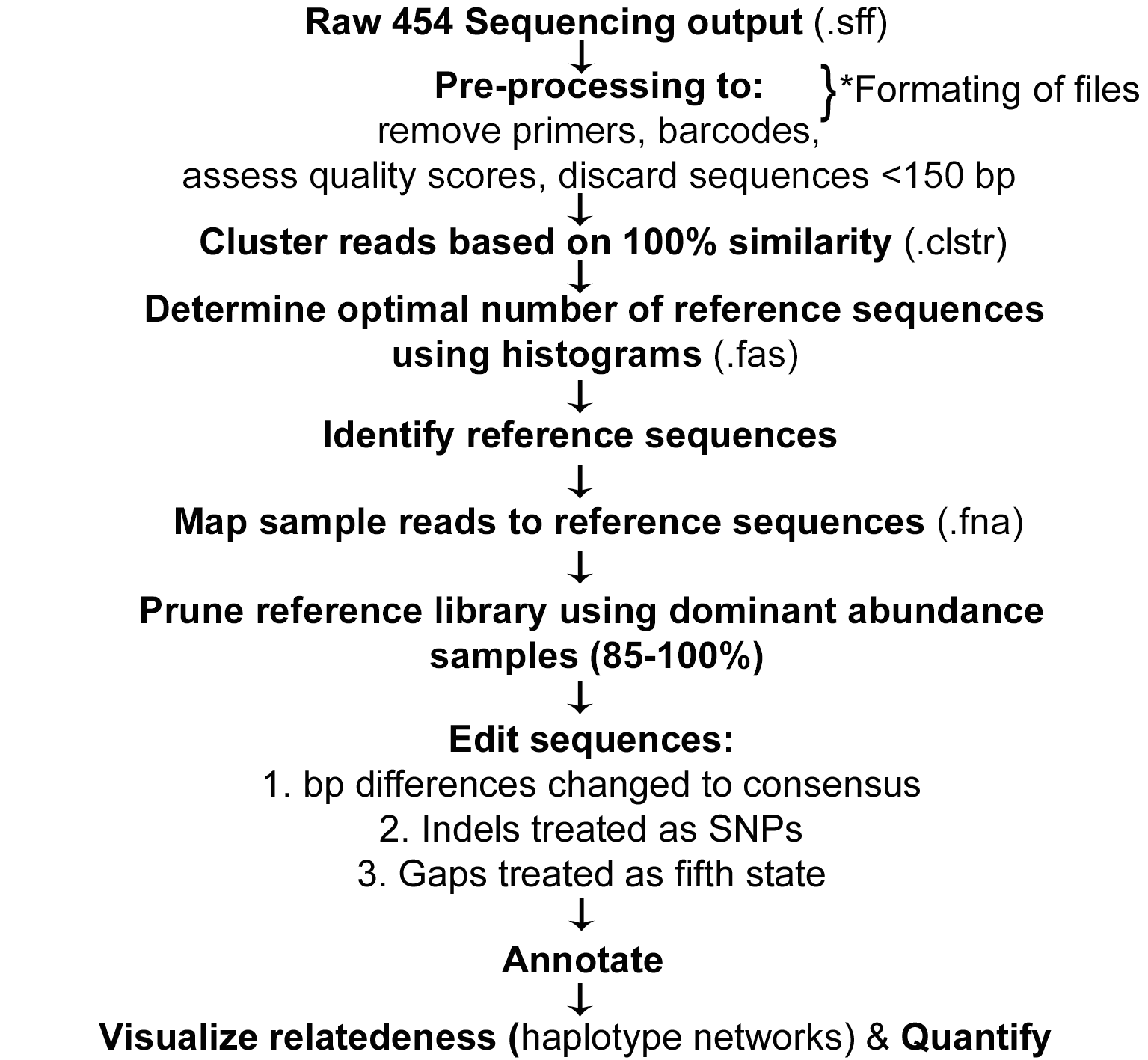

Supplement: Figure S3 — Workflow corresponding to single locus Symbiodinium 454 Next Generation Sequencing bioinformatic pipeline. *1) .sff to .fna, 2) map adaptors, trim and discard shorts, 3) convert back to .sff file incorporating this new information (trimmed .sff), 4) trimmed .sff to .fas, 5) Rename .fas to correspond to sample identities, 6) Group all renamed .fas files into one .fas file. (TIF) [file pone.0094297.s003.tif]
