# Supplementary material for: Deep-Sequencing Method for Quantifying Background Abundances of Symbiodinium Types: Exploring the Rare Symbiodinium Biosphere in Reef-Building Corals
Source: PLoS One. 2014 Apr 11;9(4):e94297. doi: 10.1371/journal.pone.0094297 (PMC3984134; doi:10.1371/journal.pone.0094297)
Supplement: File S1 — Detailed pipeline for Symbiodinium 454 Next Generation Sequencing data from a single locus (i.e. ITS-1 or ITS-2). (DOCX) [file pone.0094297.s004.docx]

**Supporting Information File S1**

**Before you begin**

The following software need to be installed and in your path.

- Cd-Hit-454 (version 0.0.2)
- 454 Sequencing System Software (version 2.5.3) {GS De Novo Assembler, GS Reference Mapper, GS Amplicon Variant Analyzer}
- R (latest version)
- Adaptors.fna (a database file containing the adaptor sequences you used)

The following Perl scripts also need to be available in your path, in addition to the scripts you’ll directly call from the command line:

- 454trim.pl
- TrimAllFNA.pl

Please contact the corresponding author for these scripts at the following email: katemarie.quigley@my.jcu.edu.au

**Main Pipeline**

The steps in this protocol can be grouped into four general tasks:

1. cleaning sequence reads

2. identifying high sequence clusters to serve as reference

3. mapping sequence reads to the reference

4. analyzing mapped reads.

If you do not have a database created for your adaptor sequences for the trimming/cleaning step you must complete the following:

1. Make an adaptors.fas file with all of the adaptor, barcodes and F and R primers
2. Type “module load blast” then “makeblastdb”
3. Type “formatdb –I adaptors.fas –p F –o T”
4. Now you are ready to run “Map the location of the sequencing adaptors and discard sequences shorter than 150bp” part of Cleaning Sequence Reads section (1.2.b)

**1. Cleaning Sequence Reads**

1. Download all raw .sff files generated from the 454 sequencing run from Fourierseq (or wherever your raw sequences were uploaded) using Secure File Transfer Protocol (SFTP) or SCP (UNIX).
2. Map the location of the sequencing primers and make sure these are removed from all reads in addition to the general quality score trimming. This step is especially important for degenerate primers.
   1. Convert raw.sff files to raw.fna files without any of the default quality trimming

> sffinfo -seq -notrim sample.sff > sample.fna

- 1. Map the location of the sequencing adaptors and discard sequences shorter than 150bp

> 454trim.pl adaptors.fna sample.fna 150 > trim.log &

c. Bring adaptor output coordinates back to original sample.sff file while maintaining original quality trimming information

> sfffile -t trimmed_sample.fna.tab -i trimmed_sample.fna.tab -o sample_trim.sff sample.sff

1. Convert all sff files with trimming info to fas

> sffinfo -seq sample_trim.sff > sample.fas

1. Rename all of the sequences in the .fas files to include some sample designation prior to concatenating all of the fasta files for cd-hit-454 analysis downstream.

> sed 's:>:>S1-:' <S1.fas >S1_new.fna

Example: This will find all “>” within the file S1.fna and will replace them with >S1 in a new file called S1_new.fna.

5. Concatenate all renamed fasta files into Alltrim.fas

> cat *new.fna > Alltrim.fas

**2. Identify high sequence clusters to serve as reference**

1. Run cd-hit to cluster reads into 100% identical groups, again with 150bp cut-off

> cd-hit-454 -c 1 -l 150 -i Alltrim.fas -o Consensus100

1. Determine number of clusters with desired number of sequences – for example, how many clusters have at least 75 sequences each; how many with 100…how many with 1000?

> grep -w 75 Consensus100.clstr

The value after –w can be changed, for example from 75 to 100, 1000, etc.

1. With the recorded num)bers in each group, make a histogram of the output using excel or R that shows the number of clusters that constitute each sequencing depth specified in step 2. These high-sequence clusters will serve as your “reference” for the subsequent mapping steps. Please see manuscript text explaining how to determine the reference cut-off.

- 1. Bring cd-hit-454 output files from the supercomputer to your computer using SFTP or SCP
  2. For the chosen cut-off limit, find the reference sequences in the primary cd-hit-454 output file using a general text-editor find. The * sequences in the .clstr file are the references for that particular cluster and searching for >O6HHJVZ1GY03FSTKJ... * in the primary output file should yield the desired unique reference sequence.
  3. Copy-paste these references into a new .fas file. Use programs like TextWrangler (MAC) or Notepad (PC).
  4. Once you have your reference.fas, you can align these sequences using your favorite sequence aligner (i.e.Seqman or BioEdit) and visually assess your clustering. Are the SNP’s logical? Are there long homopolymer runs that are indicative of 454 sequencing error? How different are the reference sequences? What do they blast to at NCBI? Are the adaptors gone?

**3. Mapping sequence reads to the reference**

1. Once you are satisfied with your reference library, bring reference.fas back to the super-computer of your choice and use it as a reference library to map individual sample files against. For each of your sequence reads run the following command:

> runMapping -rst X –o Sample reference.fas sample_trim.sff

Insert the chosen rst value where the X is located.

The –rst defaults to 12 if left unspecified. If your reference sequences are highly similar you will need to lower this threshold. Differentiating between *Symbiodinium* types required an –rst of 0. Varying this value had significant effects on the diversity of *Symbiodinium* achieved. Please see the manuscript for discussion of this value.

1. Record the number of reads mapping to each cluster for each sample. The Newbler mapper generates many output files. Use the following command to pull out the information you care about within each output folder, but you should peruse the other files to make sure there were no errors.

> grep numreads 454AllContigs.fna

1. For each sample, recorded the original number of reads, the number of reads mapped, and the number of reads mapped to each cluster.
