# Supplementary material for: Deep-Sequencing Method for Quantifying Background Abundances of Symbiodinium Types: Exploring the Rare Symbiodinium Biosphere in Reef-Building Corals
Source: PLoS One. 2014 Apr 11;9(4):e94297. doi: 10.1371/journal.pone.0094297 (PMC3984134; doi:10.1371/journal.pone.0094297)
Supplement: File S2 — Tables S1, S2, S3, and S4. (DOCX) [file pone.0094297.s005.docx]

**Table S1. ITS2 rapid-A primers used in 454 NGS sample preparation in *Symbiodinium* mixtures.** Unique barcodes used to multiplex are also included for each respective sample.

| **Name** | **Primer: A-rapid-Barcode-ITS2 reverse primer** | **Barcode** |
| --- | --- | --- |
| Ar-ITS2R-16 | CCATCTCATCCCTGCGTGTCTCCGACGACTTGTAGCGCCCTCCGCTTACTTATATGCTT | TGTAGCGC |
| Ar-ITS2R-17 | CCATCTCATCCCTGCGTGTCTCCGACGACTACACTCACCCTCCGCTTACTTATATGCTT | ACACTCAC |
| Ar-ITS2R-18 | CCATCTCATCCCTGCGTGTCTCCGACGACTACATATAGCCTCCGCTTACTTATATGCTT | ACATATAG |
| Ar-ITS2R-19 | CCATCTCATCCCTGCGTGTCTCCGACGACTACGTGATCCCTCCGCTTACTTATATGCTT | ACGTGATC |
| Ar-ITS2R-20 | CCATCTCATCCCTGCGTGTCTCCGACGACTAGTACAGACCTCCGCTTACTTATATGCTT | AGTACAGA |
| Ar-ITS2R-21 | CCATCTCATCCCTGCGTGTCTCCGACGACTAGTATCTCCCTCCGCTTACTTATATGCTT | AGTATCTC |
| Ar-ITS2R-22 | CCATCTCATCCCTGCGTGTCTCCGACGACTATACGCTGCCTCCGCTTACTTATATGCTT | ATACGCTG |
| Ar-ITS2R-23 | CCATCTCATCCCTGCGTGTCTCCGACGACTATCTAGTCCCTCCGCTTACTTATATGCTT | ATCTAGTC |
| Ar-ITS2R-24 | CCATCTCATCCCTGCGTGTCTCCGACGACTCAGCGTAGCCTCCGCTTACTTATATGCTT | CAGCGTAG |
| Ar-ITS2R-25 | CCATCTCATCCCTGCGTGTCTCCGACGACTCGCACGAGCCTCCGCTTACTTATATGCTT | CGCACGAG |
| Ar-ITS2R-26 | CCATCTCATCCCTGCGTGTCTCCGACGACTCGTGTGCGCCTCCGCTTACTTATATGCTT | CGTGTGCG |
| Ar-ITS2R-27 | CCATCTCATCCCTGCGTGTCTCCGACGACTCTAGATACCCTCCGCTTACTTATATGCTT | CTAGATAC |
| Ar-ITS2R-28 | CCATCTCATCCCTGCGTGTCTCCGACGACTCTGTCGACCCTCCGCTTACTTATATGCTT | CTGTCGAC |
| Ar-ITS2R-29 | CCATCTCATCCCTGCGTGTCTCCGACGACTTATGCACGCCTCCGCTTACTTATATGCTT | TATGCACG |
| Ar-ITS2R-30 | CCATCTCATCCCTGCGTGTCTCCGACGACTTCGCGCTACCTCCGCTTACTTATATGCTT | - |

**Table S2. ITS1 rapid-A primers used in 454 NGS sample preparation in Palau *Symbiodinium* samples.** Unique barcodes used to multiplex are also included for each respective sample.

| **Name** | **Primer: A-rapid-Barcode-ITS1 reverse primer** | **Barcode** |
| --- | --- | --- |
| Ar-ITS1R-1 | CCATCTCATCCCTGCGTGTCTCCGACGACTAGAGACTCTATCGCRCTTCRCTGCGCCCT | AGAGACTC |
| Ar-ITS1R-2 | CCATCTCATCCCTGCGTGTCTCCGACGACTAGCTCGTGTATCGCRCTTCRCTGCGCCCT | AGCTCGTG |
| Ar-ITS1R-3 | CCATCTCATCCCTGCGTGTCTCCGACGACTAGTGTCGATATCGCRCTTCRCTGCGCCCT | AGTGTCGA |
| Ar-ITS1R-4 | CCATCTCATCCCTGCGTGTCTCCGACGACTGAGCGCGCTATCGCRCTTCRCTGCGCCCT | GAGCGCGC |
| Ar-ITS1R-5 | CCATCTCATCCCTGCGTGTCTCCGACGACTGATGAGTGTATCGCRCTTCRCTGCGCCCT | GATGAGTG |
| Ar-ITS1R-6 | CCATCTCATCCCTGCGTGTCTCCGACGACTGCGAGAGATATCGCRCTTCRCTGCGCCCT | GCGAGAGA |
| Ar-ITS1R-7 | CCATCTCATCCCTGCGTGTCTCCGACGACTGCTCTCTCTATCGCRCTTCRCTGCGCCCT | GCTCTCTC |
| Ar-ITS1R-8 | CCATCTCATCCCTGCGTGTCTCCGACGACTGTCGCTGATATCGCRCTTCRCTGCGCCCT | GTCGCTGA |
| Ar-ITS1R-9 | CCATCTCATCCCTGCGTGTCTCCGACGACTGTCTAGCATATCGCRCTTCRCTGCGCCCT | GTCTAGCA |
| Ar-ITS1R-10 | CCATCTCATCCCTGCGTGTCTCCGACGACTTAGTGATATATCGCRCTTCRCTGCGCCCT | TAGTGATA |
| Ar-ITS1R-11 | CCATCTCATCCCTGCGTGTCTCCGACGACTTCACACTGTATCGCRCTTCRCTGCGCCCT | TCACACTG |
| Ar-ITS1R-12 | CCATCTCATCCCTGCGTGTCTCCGACGACTTCACTAGCTATCGCRCTTCRCTGCGCCCT | TCACTAGC |

**Table S3. Reference sequence blast identities pre- and post-editing.** N/A designates those reference sequences not used in analyses because they were not present in all high abundance samples (100-85%).

| **Reference sequence** | **Blast identity**  **without edits (E value, % Maximum identity)** | **Type designation** | **Blast identity**  **with edits (E value, % Maximum identity)** | **Type designation** | **Region** |
| --- | --- | --- | --- | --- | --- |
| A13.c | DQ174724.1 (5e-129, 95%) | B16 | DQ174724.1 (3e-83, 91%) | B16* | ITS1 |
|  | JN558096.1 (5e-84, 86% | A13 | JN558096.1 (1e-78, 81%) | A13 | 18S-ITS2, 5.8S-28S |
| A13.i | DQ174724.1 (1e-125, 94%) | B16 | N/A | - | ITS1 |
|  | EU106365.1 (4e-80, 85%) | Odo06 | N/A | - | ITS1-ITS2 |
| A13.j | DQ174724.1 (5e-124, 94%) | B16 | DQ174724.1 (3e-85, 91%) | B16 | ITS1 |
|  | JN558096.1 (7e-83, 85%) | A13 | JN558096.1 (2e-42, 81%) | A13 | 18S-ITS2, 5.8S-28S |
| A13.k | DQ174724.1 (2e-123, 94%) | B16 | DQ174724.1 (1e-83, 91%) | B16 | ITS1 |
|  | JN558096.1 (1e-80, 85%) | A13 | JN558096.1 (8e-41, 80%) | A13 | 18S-ITS2, 5.8S-28S |
| A13.l | DQ174724.1 (2e-123, 94%) | B16 | DQ174724.1 (3e-85, 91%) | B16 | ITS1 |
|  | JN558096.1 (1e-80, 85%) | A13 | JN558096.1 (2e-42, 81%) | A13 | 18S-ITS2, 5.8S-28S |
| A13.m | DQ174724.1 (2e-123, 94%) | B16 | DQ174724.1 (3e-85, 91%) | B16 | ITS1 |
|  | JN558096.1 (1e-80, 85%) | A13 | JN558096.1 (2e-42, 81%) | A13 | 18S-ITS2, 5.8S-28S |
| A13.n | DQ174724.1 (2e-123, 94%) | B16 | DQ174724.1 (3e-85, 91%) | B16 | ITS1 |
|  | JN558096.1 (1e-80, 85%) | A13 | JN558096.1 (2e-42, 81%) | A13 | 18S-ITS2, 5.8S-28S |
| A13.o | DQ174724.1 (2e-123, 94%) | B16 | DQ174724.1 (3e-85, 91%) | B16 | ITS1 |
|  | JN558096.1 (5e-84, 86%) | A13 | JN558096.1 (2e-42, 81%) | A13 | 18S-ITS2, 5.8s-28s |
| A13.p | DQ174724.1 (3e-121, 94%) | B16 | DQ174724.1 (3e-85, 91%) | B16 | ITS1 |
|  | JN558096.1 (2e-78, 85%) | A13 | JN558096.1 (2e-42, 81%) | A13 | 3.5S-ITS2, 5.8s-28s |
| A13.q | DQ174724.1 (2e-123, 95%) | B16 | DQ174724.1 (3e-85, 91%) | B16 | ITS1 |
|  | EU106365.1 (2e-78, 85%) | Odo06 | JN558096.1 (2e-42, 81%) | A13 | 18S-ITS2, 5.8s-28s |
| A13.s | DQ174724.1 (1e-90, 93%) | B16 | DQ174724.1 (3e-85, 91%) | B16 | ITS1 |
|  | JN558096.1 (7e-48, 82%) | A13 | JN558096.1 (2e-42, 81%) | A13 | 18S-ITS2, 5.8S-28S |
| A13.t | DQ174724.1 (2e-122, 94%) | B16 | DQ174724.1 (3e-85, 91%) | B16 | ITS1 |
|  | JN558096.1 (1e-79, 85%) | A13 | JN558096.1 (2e-42, 81%) | A13 | 18S-ITS2, 5.8S-28S |
| A13.u | DQ174724.1 (1e-119, 94%) | B16 | DQ174724.1 (6e-82, 90%) | B16 | ITS1 |
|  | JN558096.1 (2e-78, 85%) | A13 | JN558096.1 (5e-45, 80%) | A13 | 18S-ITS2, 5.8S-28S |
| A13.v | DQ174724.1 (2e-123, 94%) | B16 | DQ174724.1 (3e-85, 91%) | B16 | ITS1 |
|  | JN558096.1 (1e-80, 85%) | A13 | JN558096.1 (2e-42, 81%) | A13 | 18S-ITS2, 5.8S-28S |
| A13.w | DQ174724.1 (2e-118, 93%) | B16 | DQ174724.1 (1e-83, 91%) | B16 | ITS1 |
|  | JN558096.1 (4e-75, 84%) | A13 | JN558096.1 (8e-41, 80%) | A13 | 18S-ITS2, 5.8S-28S |
| A13.zz | DQ174724.1 (2e-132, 95%) | B16 | DQ174724.1 (3e-85, 91%) | B16 | ITS1 |
|  | EU106365.1 (2e-87, 86%) | Odo06 | JN558096.1 (2e-42, 81%) | A13 | 18s-28s, 5.8S-28S |
| A13.zzz | DQ174724.1 (8e-122, 93%) | B16 | DQ174724.1 (3e-85, 91%) | B16 | ITS1 |
|  | JN558096.1 (2e-78, 84%) | A13 | JN558096.1 (2e-42, 81%) | A13 | 5.8S-ITS2, 5.8s-28s |
| C1.b | JN558041.1, [JN558040.1](http://www.ncbi.nlm.nih.gov/nucleotide/344227669?report=genbank&log$=nucltop&blast_rank=2&RID=F8FC9ARF015) (2e-168, 99%) | C1 | JN558041.1, [JN558040.1](http://www.ncbi.nlm.nih.gov/nucleotide/344227669?report=genbank&log$=nucltop&blast_rank=2&RID=F8YVJPA7014)  (7e-152, 99%) | C1 | 5.8s-28s |
| C1.f | JN558041.1, [JN558040.1](http://www.ncbi.nlm.nih.gov/nucleotide/344227669?report=genbank&log$=nucltop&blast_rank=2&RID=F8FC9ARF015) (2e-162, 98%) | C1 | JN558041.1, [JN558040.1](http://www.ncbi.nlm.nih.gov/nucleotide/344227669?report=genbank&log$=nucltop&blast_rank=2&RID=F8FC9ARF015) (7e-147, 98%) | C1 | 5.8S-28S |
| C1.g | EU074889.1 (7e-167, 100%) | C1 | N/A | - | 18S-28S |
| C1.r | JN558041.1, [JN558040.1](http://www.ncbi.nlm.nih.gov/nucleotide/344227669?report=genbank&log$=nucltop&blast_rank=2&RID=F8FC9ARF015) (4e-145, 98%) | C1 | JN558041.1, JN558040.1 (3e-145 98%) | C1 | 5.8S-28S |
| C3.e | JN711498.1 (4e-160, 98%) | Clade C | JN711498.1 (3e-161, 99%) | Clade C | ITS-1-ITS-2 |
| C3.h | JN711498.1 (1e-164, 99% ) | Clade C | JN711498.1 (3e-161, 99%) | Clade C | ITS1- ITS-2 |
| C3.x | JN711498.1 (6e-168, 99%) | Clade C | JN711498.1 (5e-158, 99%) | Clade C | ITS1-ITS2 |
| D1.a | JN558076.1 (1e-165, 99%) | D1 | JN558076.1 & JN558075.1 (3e-156, 97%) | D1 | 5.8S-28S |
| D1.d | JN558076.1 (5e-159, 98%) | D1 | JN558076.1 & JN558075.1 (1e-159, 98%) | D1 | 5.8S-28S |
| D1.y | JN558080.1 (2e-163, 98%) | D1a | JN558076.1 & JN558075.1 (6e-158 , 98%) | D1 | 5.8S-28S |
| D1.z | JN558076.1 (6e-168, 99%) | D1 | JN558076.1 & JN558075.1 (1e-159, 98%) | D1 | 5.8s-28s |

******Symbiodinium* cultures were genotyped as C1, D and B16 using single-strand conformation polymorphism (SSCP) of ITS-1. Periodic genotyping of these cultures using Sanger sequencing confirmed their stability over time. Because the B16 culture was identified based on ITS-1 ([DQ174724.1](http://www.ncbi.nlm.nih.gov/nucleotide/86562571?report=genbank&log$=nucltop&blast_rank=1&RID=F3J2KM4Z015)) and later annotated as A13 with sequencing of the ITS-2 marker, it is here referred to as A13 (see Methods: f. Assigning *Symbiodinium* type using blast for more detail).

**Table S4. Summary of robust gamma rank correlation tests for C1, C3 and A13 haplotypes of percent observed data.** Note: *p* values may vary slightly as this value is calculated using the random shuffling of 1000 values. *Significant *p* values

| **Pair-wise haplotype (h) comparisons** | **Gamma statistic** | **Significance value** |
| --- | --- | --- |
| C3 (h) x C3 (e) | 0.72 | 0.002* |
| C3 (h) x C3 (x) | 0.76 | 0.002* |
| C3 (e) x C3 (x) | 0.42 | 0.092 |
| C1 (b) x C1 (f) | 0.84 | 0.001* |
| C1 h (b) x C1 (r) | 0.77 | 2.2 x 10-16* |
| C1 h (f) x C1 (r) | 0.84 | 0.003* |
| A13 (c) x A13 (j) | 0.93 | 0.002* |
| A13 (c) x A13 (k) | 0.95 | 2.2 x 10-16* |
| A13 (c) x A13 (l) | 0.93 | 0.002* |
| A13 (c) x A13 (m) | 0.93 | 0.004* |
| A13 (c) x A13 (n) | 1 | 2.2 x 10-16* |
| A13 (c) x A13 (o) | 0.86 | 0.002* |
| A13 (c) x A13 (p) | 0.85 | 0.001* |
| A13 (c) x A13 (q) | 0.96 | 0.004* |
| A13 (c) x A13 (s) 10 | 0.95 | 0.001* |
| A13 (c) x A13 (t) 11 | 0.96 | 2.2 x 10-16* |
| A13 (c) x A13 (u) | 0.92 | 2.2 x 10-16* |
| A13 (c) x A13 (v) | 0.97 | 2.2 x 10-16* |
| A13 (c) x A13 (w) | 0.93 | 2.2 x 10-16* |
| A13 (c) x A13 (zz) | 0.98 | 2.2 x 10-16* |
| A13 (c) x A13 (zzz) | 0.88 | 2.2 x 10-16* |
| A13 (j) x A13 (p) | 0.87 | 0.001* |
| A13 (j) x A13 h (q) | 0.91 | 0.088* |
| A13 (k) x A13 (p) | 0.81 | 0.002* |
| A13 (k) x A13 (q) | 0.91 | 0.033* |
| A13 (n) x A13 (p) | 0.85 | 0.001* |
| A13 (u) x A13 (q) | 0.86 | 0.063 |
| A13 (p) x A13 (u) | 0.78 | 0.004* |
| A13(p) x A13 (zzz) | 0.73 | 0.005* |
| A13(w) x A13 (u) | 0.85 | 0.001* |
| A13(w) x A13(zzz) | 0.81 | 2.2 x 10-16* |
| A13 (zzz) x A13 (u) | 0.90 | 0.002* |
| A13 (zzz) x A13 (w) | 0.81 | 0.002* |
